# Molecular dissection of the oncogenic role of ETS1 in the mesenchymal subtypes of head and neck squamous cell carcinoma

**DOI:** 10.1371/journal.pgen.1008250

**Published:** 2019-07-15

**Authors:** Christian Gluck, Alexandra Glathar, Maria Tsompana, Norma Nowak, Lee Ann Garrett-Sinha, Michael J. Buck, Satrajit Sinha

**Affiliations:** Department of Biochemistry, SUNY at Buffalo, Buffalo, NY, United States of America; Novartis, UNITED STATES

## Abstract

Head and Neck Squamous Cell Carcinoma (HNSCC) is a heterogeneous disease of significant mortality and with limited treatment options. Recent genomic analysis of HNSCC tumors has identified several distinct molecular classes, of which the mesenchymal subtype is associated with Epithelial to Mesenchymal Transition (EMT) and shown to correlate with poor survival and drug resistance. Here, we utilize an integrated approach to characterize the molecular function of ETS1, an oncogenic transcription factor specifically enriched in Mesenchymal tumors. To identify the global ETS1 cistrome, we have performed integrated analysis of RNA-Seq, ChIP-Seq and epigenomic datasets in SCC25, a representative ETS1^high^ mesenchymal HNSCC cell line. Our studies implicate ETS1 as a crucial regulator of broader oncogenic processes and specifically Mesenchymal phenotypes, such as EMT and cellular invasion. We found that ETS1 preferentially binds cancer specific regulator elements, in particular Super-Enhancers of key EMT genes, highlighting its role as a master regulator. Finally, we show evidence that ETS1 plays a crucial role in regulating the TGF-β pathway in Mesenchymal cell lines and in leading-edge cells in primary HNSCC tumors that are endowed with partial-EMT features. Collectively our study highlights ETS1 as a key regulator of TGF-β associated EMT and reveals new avenues for sub-type specific therapeutic intervention.

## Author summary

The expression of the transcriptional regulator, E26 transformation-specific 1 (ETS1), is elevated in many epithelial cancers and portends aggressive tumor behavior and poor survival. Within these carcinomas, ETS1 function has been shown to be associated with a wide range of cellular responses that include increased proliferation, angiogenesis, metastasis and drug resistance. Here we focus on Head and Neck Squamous Cell Carcinoma (HNSCC) and discover that higher expression of ETS1 is specifically more pronounced in the mesenchymal subtypes of HNSCC, which represent tumors with enriched expression of Epithelial to Mesenchymal Transition (EMT) markers and inflammation. By using genomic and epigenomic strategies, we have identified the global targets of ETS1 in a preclinical Mesenchymal HNSCC cell model and determined the crucial gene network that is most dependent upon its function. We further validate this ETS1-driven gene expression signature within several HNSCC patient derived datasets and conclude that ETS1 acts as a crucial regulator of the TGFβ signaling cascade to drive EMT. Our findings reinforce the challenges of epithelial tumor heterogeneity and offer insights into molecular underpinning of a specific subtype that can be mined for cancer vulnerability.

## Introduction

Head and neck squamous cell carcinoma (HNSCC) is the sixth most common cancer world-wide, with an annual incidence of roughly 600,000 cases; 50,000 cases are diagnosed annually in the United States alone[1, 2]. HNSCC carries a 5-year mortality rate of nearly 50%, making it a leading cause of cancer-related death[3]. The major etiologies of this cancer are related to tobacco and alcohol consumption as well as infection with human papillomavirus (HPV), which has emerged in the past two decades as a prominent risk factor leading to a substantial proportion of HNSCCs diagnosed each year[4–8]. Common treatment regimens for HNSCC include surgery, radiation and cytotoxic chemotherapy. However, in the past 15 years, survival has not improved significantly, especially in patients with advanced and recurrent disease, which highlights the need for better understanding of the disease and personalized therapies [6, 9].

A challenging aspect of HNSCC biology is that it is inherently a diverse disease, comprised of tumors from distinct anatomical locations spanning the entire upper aerodigestive tract[8]. In addition, HNSCC presents with enormous variability in patient prognosis as well as response to therapy, which may denote an underlying molecular heterogeneity[10, 11]. Indeed, recent genomic sequencing studies of HNSCC have revealed this complex heterogeneity, and importantly also highlighted the lack of consensus and actionable driver mutations[12–16]. Initial grouping of HNSCC patients based on the unsupervised clustering of global gene expression revealed four major subtypes that were designated as Basal, Mesenchymal, Classical and Atypical[17, 18]. However, the generation of new datasets, particularly those enriched in epigenomic features and the application of advanced bioinformatics tools for meta-analysis have begun to offer us a more detailed view of the various HNSCC molecular subtypes[19–21]. Importantly, it is becoming clear that each subtype of HNSCC possess a distinct pattern of gene expression that sets it apart in terms of the underlying biology, key drivers and potentially actionable targets.

Notwithstanding the emerging nuances of the clustering profiles of HNSCC, the Mesenchymal subtype of HNSCC remains a persistent entity across various datasets and warrants further investigation due to its aggressive behavior. As implied in its name, the mesenchymal subtype of tumors show enriched expression of key drivers of the Epithelial-Mesenchymal Transition (EMT) and those associated with functional pathways of cell motility and angiogenesis[22–24]. Of note, HNSCC tumors characterized by elevated levels of classical EMT markers exhibit high rates of distant metastasis and shorter metastasis-free survival[25, 26]. The distinct dysregulated gene expression programs which control phenotypes of the various subtypes of HNSCC, including the ETS1^high^ mesenchymal, are likely to be driven by specific transcriptional regulators[27]. However, our current knowledge of the identity of such factors and understanding of their mechanisms of action remain sparse.

Here, we have utilized a comprehensive epigenomic and transcriptomic approach to uncover a novel regulator of the Mesenchymal subtype of HNSCC. We show that ETS1, an oncogenic transcription factor (TF) and prototypic member of the ETS family of TFs, is preferentially expressed in Mesenchymal HNSCC across several large-scale transcriptomic datasets. We comprehensively probed the molecular function of ETS1 within a Mesenchymal HNSCC cell-line model, SCC25, by first identifying its direct target genes using a combination of ChIP-Seq analysis, and RNA-Seq analysis. Our studies uncovered a core ETS1 driven Mesenchymal gene signature, which identifies select genes that are highly correlative with the EMT process. Significantly, we observed that one major role of ETS1 could be in driving and maintaining the Mesenchymal state of HNSCC through the TGF-β signaling cascade. Our findings have likely relevance for human HNSCC tumors as exemplified by an enriched ETS1 gene signature in subpopulations of cells exhibiting a partial-EMT state that have been uncovered by recent single cell RNA-Seq (scRNA-Seq) analysis[28].

## Results

### ETS1 is overexpressed in head and neck squamous cell carcinoma and associated with poor survival

**Fig 1:**
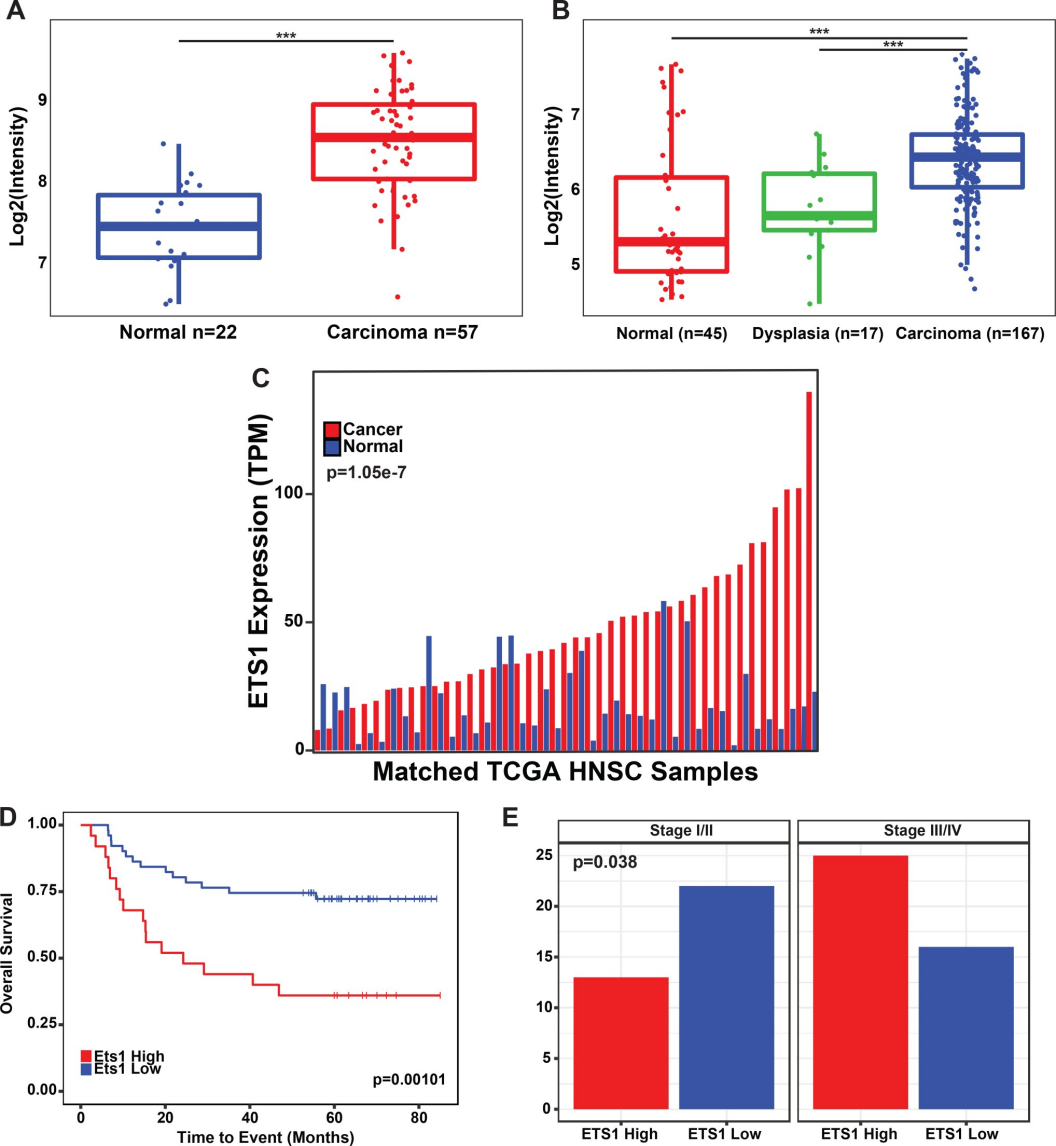
ETS1 is highly expressed in HNSCC tumors and is associated with poor patient survival. Boxplots displaying the distribution of ETS1 probe intensity, across Normal, Dysplastic and Cancerous Tissue, in the **(A)** Peng et al. (2011), (p = 5.16e-10) and the **(B)** Chen et al. (2008) microarray datasets, (p = 3.6e-6, p = 2.46e-4). **(C)** Bar-graph displaying the expression of ETS1 in the HNSC TCGA dataset. Transcriptomic data is from tumor samples with matched normal mucosa (n = 43) The bar-graph is displayed in ascending order (left-right) based on the expression levels of ETS1. **(D)** Kaplan-Meier plot of the overall survival of patients in the Chen et al. (2008) dataset (n = 97), based on the expression of ETS1 (ETS1 High, n = 49, ETS1 Low, n = 48). **(E)** Bar-graph displaying the distribution of HNSCC clinical stages based on the expression of ETS1 in the Chen et al. (2008) dataset, (p = 0.038, Chi-Square). X-axis denotes the number of cases.

Although an oncogenic role of ETS1 in HNSCC has been previously reported, such studies have been limited in scope predominantly to immunostaining of tumors from a relatively small number of patients[29–31]. Therefore, here, we examined available transcriptomic datasets to re-assess the ETS1 expression profile in various stages of HNSCC [14, 32, 33]. Our analysis revealed that across multiple microarray (Fig 1A and 1B) and RNA-Seq (Fig 1C) based datasets, ETS1 mRNA levels were consistently high in tumors compared to normal tissues and interestingly, showed increasing expression as tumors progressed from dysplastic to carcinoma states. The tumor-enriched expression of ETS1 was most strikingly discernible in the RNA-Seq data from the Cancer Genome Atlas (TCGA), where ETS1 levels were higher in ~80% of the patient tumor samples compared to matched normal tissue (Fig 1C). The high levels of ETS1 was associated with poor prognosis as evident from Kaplan-Meier analysis of a cohort of 97 HNSCC patients, which showed that patients with high ETS1 expression had a statistically significant overall worse outcome than patients with low expression (Fig 1D). While patients with low ETS1 expression had a median overall survival time of 61 months, the median overall survival time of patients with high levels of ETS1 expression was reduced to 34 months. Furthermore, ETS1 expression in these patients was shown to significantly correlate with higher clinical stage (Fig 1E) with higher levels specifically associated with the late stages (III/IV) of cancer.

### ETS1 is enriched in the mesenchymal subtype of HNSCC and is a core component of the EMT signature

**Fig 2:**
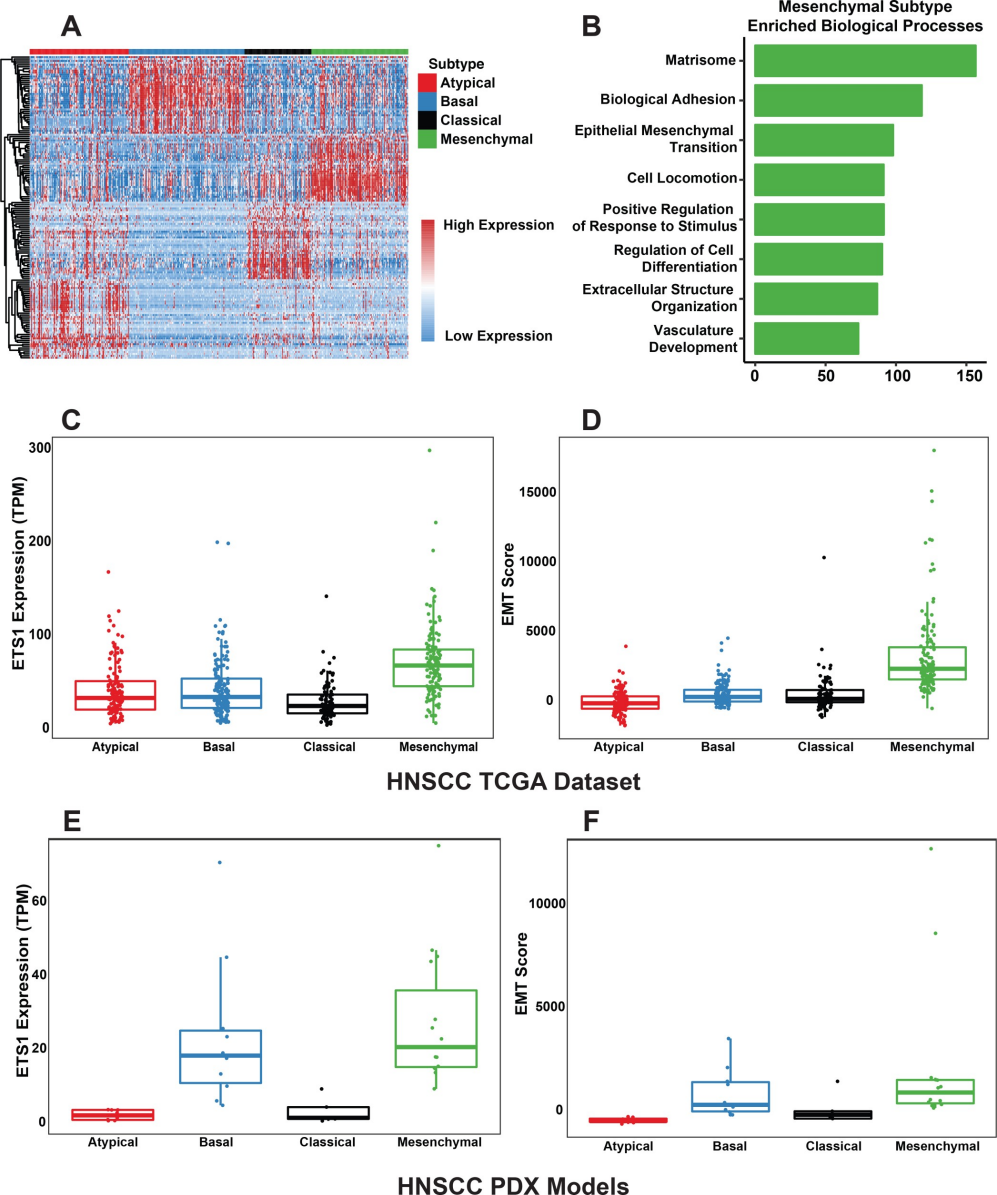
ETS1 is enriched in the EMT^high^ Mesenchymal subtype of HNSCC across various datasets. **(A)** Heatmap displaying the hierarchical clustering of the expression values of the top 50 genes specifically enriched in each subtype present in the TCGA HNSCC Dataset. **(B)** Bargraph highlighting enriched Biological Processes (GO Consortium) in Mesenchymal HNSCC as determined by the subtype specific DESeq2 analysis (Hypergeometric Test, FDR < 0.05). X-axis: -log(Adjusted p-value). **(C)** Boxplots displaying the HNSCC subtype-specific distribution of ETS1 expression and **(D)** EMT score in the TCGA HNSCC dataset. **(E)** Boxplots displaying the HNSCC subtype-specific distribution of ETS1 expression and **(F)** EMT score in the HNSCC PDX dataset.

The molecular heterogeneity of HNSCC is clearly evident by the four major gene expression subtypes that have been derived from clustering analysis of both microarray and RNA-Seq datasets generated from tumor patients[14, 17, 32, 33]. We sought to both confirm these findings as well as generate an improved subtype specific gene-signature by performing differential gene expression analysis (DEG) on the updated and much larger TCGA RNA-Seq dataset of 504 HNSCC tumors. Hierarchical clustering analysis based on the mRNA expression level of the most highly expressed subtype-specific genes reaffirmed the existence of the four defined subtypes—Atypical (26.2%), Basal (30.6%), Classical (17.6%), and Mesenchymal (25.6%) (Fig 2A). As expected, these subtypes were associated with biological functional pathways and processes characteristic of their distinct molecular and cellular attributes. Indeed, gene ontology enrichment analysis of the Mesenchymal (MS) subtype tumors highlighted several processes associated with EMT such as the remodeling of the ECM, cellular locomotion, cellular differentiation in addition to EMT itself (Fig 2B, S1 Table, MSigDB Biological Processes) [34, 35]. In contrast, examination of the ontology enrichments for the other three intrinsic subtypes yielded results that matched closely to the original findings from the subtype analysis [19, 36] (S1 Fig, S1 Table). Interestingly, we observed a significant enrichment of ETS1 expression (Fig 2C) in the MS subtype whereas a closely related family member, ETS2 did not show any sub-type specific expression pattern (S1 Fig).

To examine how well ETS1 expression correlates with EMT, we next used a recently devised robust EMT scoring method [37]to assess and rank each MS subtype tumor based on their level of EMT. As illustrated in a heatmap highlighting the range of EMT and the expression levels of selected subset of key EMT-related markers, tumors with the highest EMT score were also associated with the highest levels of ETS1 expression (S2A Fig). Notably, examination of the distribution of the EMT score across the 4 subtypes of HNSCC clearly showed the MS subtype to have much higher level of enrichment (Fig 2D). To validate the findings from this analysis, we examined the respective protein levels of EMT-related genes in HNSCC tumors derived from the TCGA Reverse Phase Protein Array dataset (RPPA) [38]. We observed similar overall trends in the protein and mRNA transcript levels of the genes shared between the RPPA and RNA-Seq datasets, as well as strong correlation of ETS1 protein with EMT Score, in agreement with the ETS1 mRNA level (S2B Fig).

One challenge of working with the available TCGA datasets is the confounding effects of tumor purity on the correlating and clustering analysis of global gene expression. To address this limitation, we next examined RNA-Seq data from 38 HNSCC Patient Derived Xenografts (PDX) [39]. We reasoned that the PDX datasets are likely to offer a more tumor-centric analysis due to the fact that the human stromal component of PDX models is often lost in early passages[40] and that the mouse stromal contribution can be eliminated during downstream RNA-Seq processing. Interestingly, even with a relatively small sample size, gene expression profile (Fig 2E) in the HNSCC PDXs mirrored the established distribution of the HNSCC subtypes (21% Atypical, 26.3% Basal, 13.2% Classical and 39.5% Mesenchymal) and importantly, ETS1 expression was preferentially enriched in Mesenchymal subtype (Fig 2F) similar to TCGA datasets. Taken together, our analysis across multiple platforms revealed that ETS1 expression is preferentially enriched in the Mesenchymal subtype of HNSCC and it is highly correlated with the EMT score of tumors.

### Mapping the genomic targets of ETS1 in SCC25, a prototypic MS subtype of HNSCC cell line

**Fig 3:**
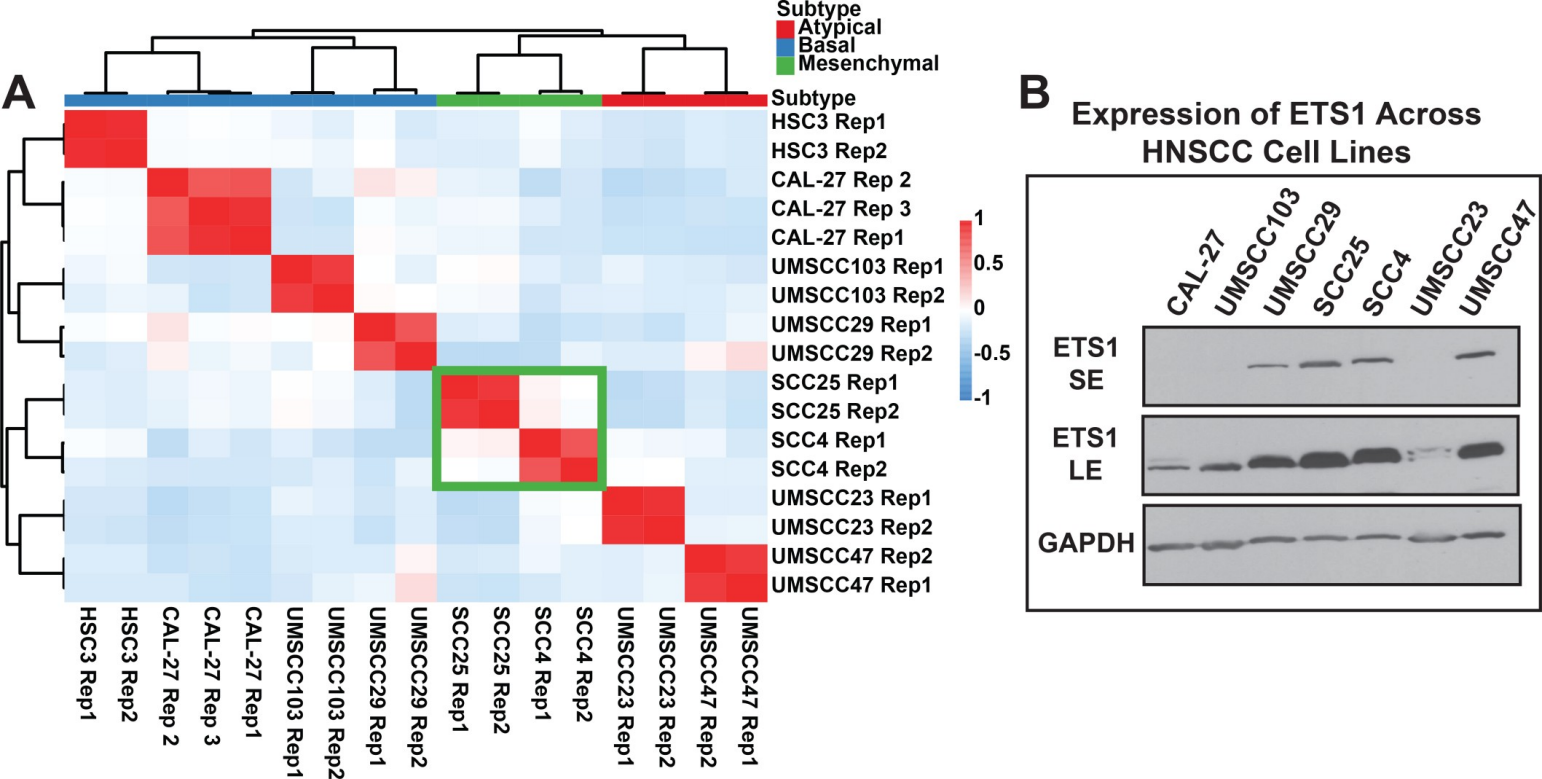
SCC25, a representative HNSCC cell line expresses high levels of ETS1. **(A)** Heatmap showing the cross-correlation value of the top 1500 most variably expressed genes in 8 HNSCC cell lines of different subtypes. The correlation matrix was reorganized via hierarchical clustering (Pearson Correlation, complete linkage). The green box highlights the two Mesenchymal classified cell lines, SCC25 and SCC4. **(B)** Western blot analysis of ETS1 across HNSCC cell lines. SE, short exposure; LE, long exposure; GAPDH, loading control.

For follow up studies to our observations from tumor datasets and to identify a suitable cell line for downstream function analysis, we next performed RNA-Seq analysis of eight representative HNSCC cell lines. Unsupervised hierarchical clustering analysis of gene expression profiles of the HNSCC cells allowed us to categorize them based on the intrinsic subtype classification (Fig 3A), with two of the tongue-derived SCC cell lines, SCC4 and SCC25 matching the MS subtype. The SCC25 cell line, in particular exhibited the highest level of expression of ETS1 mRNA and importantly, the most enriched EMT score (S2C Fig). The transcriptomic data based findings were corroborated by Western blot analysis with anti-ETS1 antibodies which confirmed relatively higher levels of ETS1 protein in the SCC25, compared to other cell lines (Fig 3B). The observation that the HNSCC cell lines retain subtype-specific gene expression profiles prompted us to further validate this in additional publicly available datasets. Similar analysis of RNA-Seq data of HNSCC cells generated by the Cancer Cell Line Encyclopedia Project as well as those from a recently published study[41, 42] yielded similar results, whereupon cell lines of a specific subtype clustered together and segregated into four distinct molecular groups (S3 Fig). The SCC25 cell line again showed high levels of ETS1 and a EMT-high mesenchymal gene expression program in these two broad datasets and hence was chosen for downstream analysis of ETS1 function.

**Fig 4:**
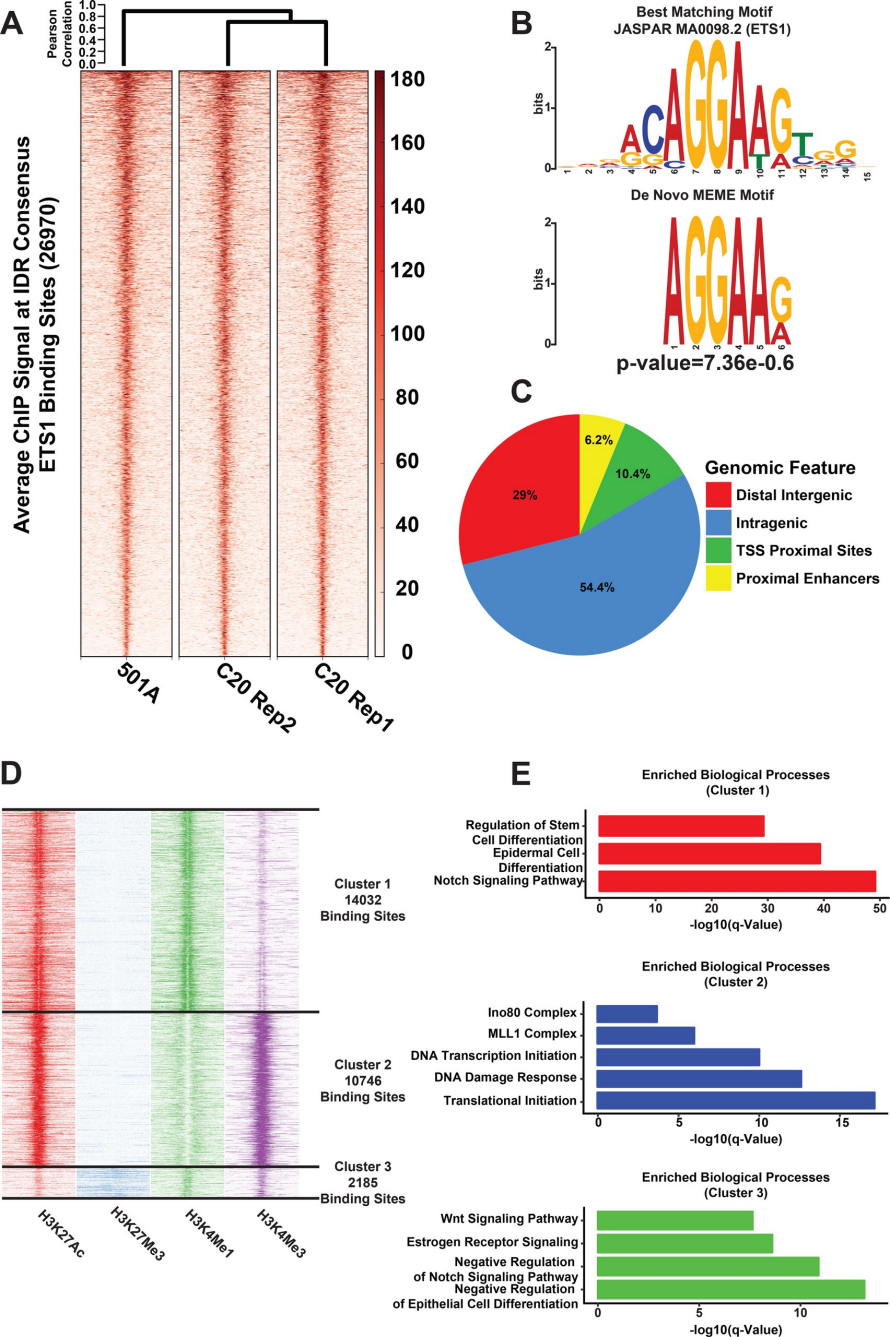
ChIP-Seq analysis reveals direct ETS1 targets and their epigenomic features in SCC25 cells. **(A)** Heatmap of the ChIP-Seq signal at the IDR-generated consensus ETS1 binding sites across three ChIP replicates. The individual ChIP-Signal matrices were subjected to hierarchical clustering and presented as a dendrogram **(B)** The top de novo motif, derived from the ETS1 ChIP-Seq peak-set as generated by MEME. The most similar JASPAR motif (2018 Database) as determined by TOMTOM analysis is shown above the MEME motif. **(C)** Distribution pattern of ETS1 binding sites across the genome. **(D)** Heatmap of histone modification signal density using k-means clustering on ETS1 binding sites showing three different groups. **(E)** Bargraphs displaying selected top enriched gene-sets (GO Biological Processes and MSigDB pathways) associated with genes annotated to 3 clusters of ETS1 ChIP-Seq peaks.

To examine the molecular mechanisms of ETS1 function, we next identified its global DNA-binding sites in SCC25 cells by ChIP-Seq experiments using two different anti-ETS1 antibodies. By using the ENCODE recommended IDR (Irreproducible Discovery Rate) method for data analysis, we identified 26,760 ETS1 genomic-bound sites that showed a high correlation among the ChIP-Seq replicates of three independent experiments (Fig 4A, S2 Table). As expected, de novo motif analysis using MEME revealed the core ETS DNA-Binding motif as the most enriched motif in the ETS1-bound sites. This was further verified by TOMTOM based motif comparison, which revealed that the ChIP-Seq derived motif was nearly identical to the ETS1 DNA-Binding Motif (p = 7.36e-6) [43] (Fig 4B). The distribution of the ETS1 peaks relative to transcription start sites (TSS) showed that ETS1 preferentially bound to distal regulatory regions with the majority of sites being classified as intragenic (Fig 4C).

### ETS1 binding in context of the epigenomic landscape of SCC25 and its preferential targeting of Super-Enhancers

We investigated the ETS1-genomic interactions in the chromatin context by analyzing the histone modification profile around each individual ETS1 binding site, using H3K27Ac, H3K27Me3, H3K4Me1, and H3K4Me3 ChIP-Seq data from the SCC25 cell line. This cohort of histone marks, in various combinations can be used to identify active enhancers (H3K27Ac^High^:H3K4Me1^High^), active promoters (H3K27Ac^High^:H3K4Me3^High^) and poised enhancers (H3K27Me3^High^:H3K4Me1^High^:H3K27Ac^Low^) [44]. By calculating the normalized levels of histone modification ChIP-Seq signal at each ETS1 bound genomic region and performing k-Means clustering of differentially enriched epigenetic profiles, we identified three clusters of ETS1 binding. (Fig 4D, S3 Table). While most of the ETS1 binding overlapped with active regulatory elements characterized by the tell-tale histone modification features of enhancers and promoters, a third distinct cluster of ETS1 binding was found to exhibit epigenetic features of bivalent and poised chromatin domains. Notably, these 3 clusters of ETS1 bound genomic regions were associated with genes associated with different enriched biological processes (Fig 4E). The top enriched pathways for genes in the cluster 1 was for the maintenance of stem and epidermal cell differentiation, whereas genes associated with cluster 2 were associated with house-keeping functions such as transcriptional initiation as well as chromatin organization. Interestingly, cluster 3 represented genes involved in the negative regulation of epithelial cell differentiation, as exemplified by the enrichment of genes involved in the inhibition of Notch signaling pathway. The interesting observation that ETS1 shows a contrasting bias of enriched binding to promoters for house-keeping genes and to distal enhancers that are likely to drive tissue-specific gene expression programs is consistent with previous ETS1 ChIP-seq results from other cell types[45]. Overall, this analysis suggested a global role of ETS1 as a transcriptional regulator of the cellular stem and differentiation state of SCC25 cells in particular, and by extension possibly other epithelial cancer cells in general.

**Fig 5:**
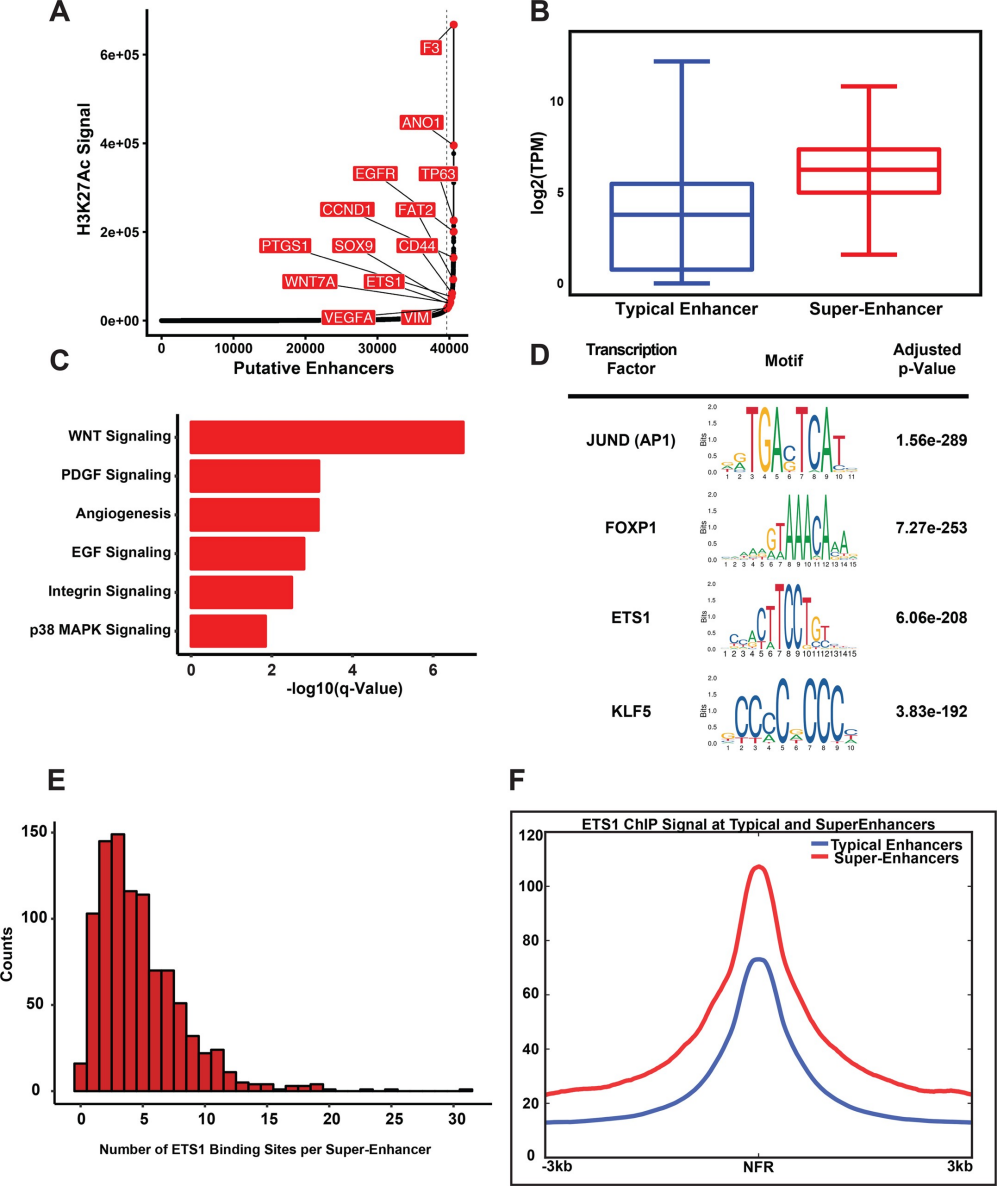
Super-Enhancers in SCC25 and enriched binding of ETS1. **(A)** Line plot displaying the ranked H3K27Ac ChIP-Seq signal in SCC25. Representative genes marked by Super-Enhancer (SE) are shown. **(B)** Boxplot of RNA-Seq expression values of genes associated with typical enhancers and Super-Enhancers. **(C)** Bargraph of SE marked top enriched MSigDB pathway. **(D)** Top enriched DNA-Binding motifs of transcription factors found within the Nucleosome Free Regions (NFR) of SCC25 SEs. **(E)** The distribution of binding events of ETS1 with SEs displayed as a histogram. **(F)** Linegraph showing the differences in the average ETS1 ChIP-Seq signal measured at Nucleosome Free Regions present in either SEs or typical enhancers.

The dynamic interaction of ETS1 with distinct chromatin states prompted us to next examine Super-Enhancers (SEs) that are typically populated by master TFs and have the highest content of the active chromatin mark H3K27ac[46–48]. We generated the SE landscape of SCC25 by implementing the Rose algorithm with the H3K27Ac ChIP-Seq data and identified 965 such regulatory elements (S4 Table). As expected, several SEs are associated with master regulators and critical components of epithelial development and differentiation, such as EGFR and TP63, in agreement with the squamous origin of the SCC25 cell line (Fig 5A, S4 Fig). Additional SE-marked genes included those related to stem cell biology and EMT, such as SOX9 and Vimentin and importantly, ETS1 itself (Fig 5A, S4 Fig). Interestingly, in keeping with the functional importance of SEs, SE-associated genes exhibit higher median expression compared to those associated with Typical Enhancers (TEs) and show enrichment of signaling pathways and processes linked to oncogenic hallmarks such as angiogenesis and the p38 MAPK signaling, in addition to pathways associated with EMT, such as PDGF and WNT signaling (Fig 5B and 5C).

To identify potential TFs that might preferentially bind to SEs, we next performed motif enrichment analysis of nucleosome free regions (NFRs) within the SCC25 SEs. By using the motif enrichment tool AME and querying the JASPAR motif database [49], we found that the ETS motif was one of the most-enriched motif based on multiple hypothesis adjusted p-value, along with AP-1, a known co-factor[50] of ETS proteins (Fig 5D). Indeed, compared to TEs, where only a quarter were bound by ETS1, almost all SEs (98%) were occupied by ETS1, with dense ETS1 binding of an average of nearly 5 times per SE (Fig 5E). This preferential association of ETS1 to SEs is further evident when the average ETS1 ChIP-Seq signal per NFR was compared between SEs and TEs (Fig 5F). Taken together, these findings suggest preferred binding of ETS1 in the SEs of SCC25 as a mode to regulate various important cellular facets of HNSCC.

### Loss of ETS1 Inhibits SCC25 and SCC4 cellular growth and invasion

**Fig 6:**
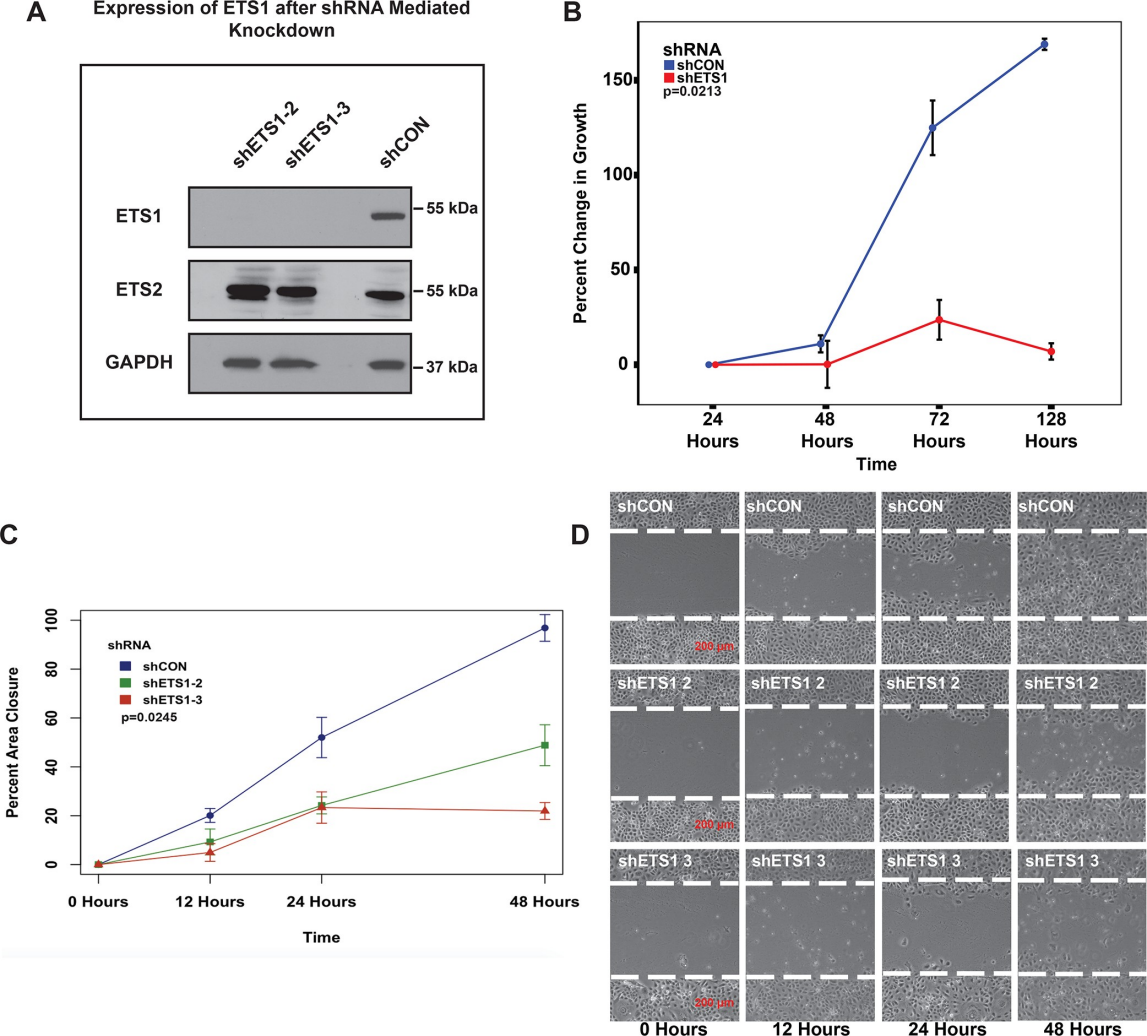
Loss of ETS1 affects SCC25 cell proliferation and migration. **(A)** Western Blot analysis of expression of ETS1 and ETS2 in SCC25 cells either expressing either ETS1-targeting shRNAs or a non-targeting shRNA (shCON). GAPDH, loading control **(B)** Line plot displaying the differences in cellular proliferation between SCC25-shETS1 and SCC25-shCON cells, as determined by the MTT assay, (p = 0.0213, ANOVA, Tukey Post-Hoc Test). Wound scratch-healing assays for assessing cell migration after mitomycin treatment. **(C)** Percent area closure of the initial wound area of the SCC25-shETS1 and SCC25-shCON cells. **(D)** Representative images of the wound area after 0, 24 and 48 hours are displayed in the right (p = 0.0318, ANOVA, Tukey Post-Hoc). White hash marks denote the boundary of the wound.

In order to investigate the functional relevance of ETS1 and to identify its target genes, we next undertook knockdown (kd) studies. Towards this end, we used a lentiviral based system to generate SCC25 cells that stably express previously validated ETS1-targeting shRNAs [51] or a control, non-targeting shRNA (shCON). Efficient and specific knockdown of ETS1 was achieved by two independent shRNAs, sh2 and sh3, as evident by western blot analysis which showed robust loss of ETS1 expression but no effect on closely related ETS2 protein (Fig 6A). Based on results obtained from several independent experiments, ETS1kd by sh3 was more robust and consistent, in agreement with published results [51]. Hence for most of the subsequent studies, SCC25 stable cells with sh3-mediated knockdown were utilized and independent verification of key findings were performed by ETS1kd by sh2, wherever necessary. Reduced ETS1 expression and/or overexpression of ETS1 in other cell lines, including prostate and breast cancer cells have been previously shown to influence cell growth and migration[52–55]. To examine if ETS1 behaved similarly in SCC25 cells, we examined the effect of the loss of ETS1 using a MTT proliferation assay. A significant reduction in percent cell growth was observed in the cells expressing the ETS1kd compared to the control (Fig 6B). To follow-up on these observations, we assessed the requirement of ETS1 for the SCC25 cells to migrate and invade in both 2D and 3D cell culture model systems. We first used the wound healing scratch assay to assess the ability of a confluent mono-layer of the stably transfected SCC25 cells to invade into identical denuded, cell-free areas within a cell culture plate. To negate the confounding effects of the cell proliferation defects upon loss of ETS1, we performed the wounding experiment after treating the cells with mitomycin, a proven cell growth inhibitor. Loss of ETS1 greatly inhibited the ability of SCC25 cells to migrate into the cell-free zone since after 48 hours, while the control cells completely closed the wound, the ETS1-depleted cells managed to invade only ~20% (sh3) or 40% (sh2) of the area (Fig 6C). Next, we tested the ability of ETS1-depleted SCC25 cells to invade through 3D matrix by using the Boyden chamber invasion assay. The control SCC25 cells had nearly 7 times more cells invade as compared to the SCC25-ETS1kd cells (S5 Fig). To confirm these findings, we next decided to perform similar independent experiments with the SCC4 cell line, which is not only mesenchymal in its gene expression traits but also has high expression of ETS1. Stable SCC4 cells were established for ETS1 knockdown by shRNAs, sh2 and sh3 as in SCC25 cell line. The effects of loss of ETS1 was more pronounced in SCC4 cell line such that the cells infected with the more potent sh3 were severely growth arrested and could not be propagated long term in culture easily. However, MTT proliferation and wound healing scratch assays with SCC4 control and ETS1sh2kd cells recapitulated the findings of SCC25 cells with loss of ETS1 leading to inhibition of cell growth and migratory capacity, respectively (S6 Fig). Taken together, these results confirmed previous findings and supported the notion that ETS1 plays an important role in the growth, proliferation and migratory potential of the HNSCC mesenchymal cells.

### Loss of ETS1-induced gene Expression leads to disrupted TGFB signaling

**Fig 7:**
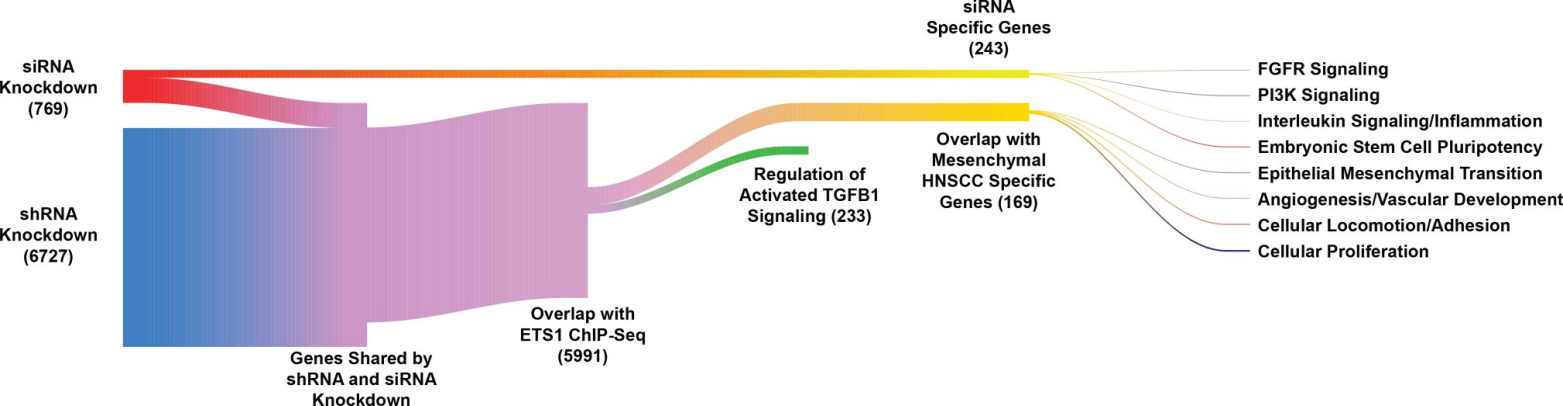
Identification and analysis of global ETS1 target genes. Sankey plot showing target genes of ETS1 based on the integration of RNA-Seq and ChIP-Seq data-sets. The tails of the plot display selected ETS1-dependent pathways revealed by gene-set enrichment analysis.

As a means to identify ETS1 targets, we next performed RNA-Seq experiments to profile the global transcriptomic changes that are unleashed upon loss of ETS1. For these studies, we employed both siRNA transfected SCC25 cells and the previously described shRNA transduced SCC25 stable cell lines to examine the short and long term effects of ETS1 loss, respectively. As to be expected from prolonged loss of ETS1, shRNA mediated ETSkd resulted in a significant alteration in the mRNA landscape of the SCC25 cells with 6727 genes showing statistically meaningful changes (S5 Table). In contrast, the changes associated with the siRNA mediated knockdown were modest, resulting in 786 genes with significant up or down regulation despite the marked reduction in the levels of ETS1 transcripts in the ETS1-siRNA treated cells. Interestingly, there was a 67% overlap in Differentially Expressed Genes (DEGs) profile between the two experimental conditions (S5 Table). This raised the possibility that some of the effects of loss of ETS1 are transitory in nature–indeed there were 243 genes that were altered only in the siRNA experiment. This cohort of DEGs that were exclusive to the siRNA-mediated knockdown of ETS1 were involved in PI3K signaling, as well as processes that have been associated with EMT in cancer such as inflammation and FGF signaling. A number of altered genes also included members of the WNT and Notch signaling cascades, which are involved in the maintenance of embryonic stem cell pluripotency and have also been linked to EMT and cancer stem cells (Fig 7).

**Fig 8:**
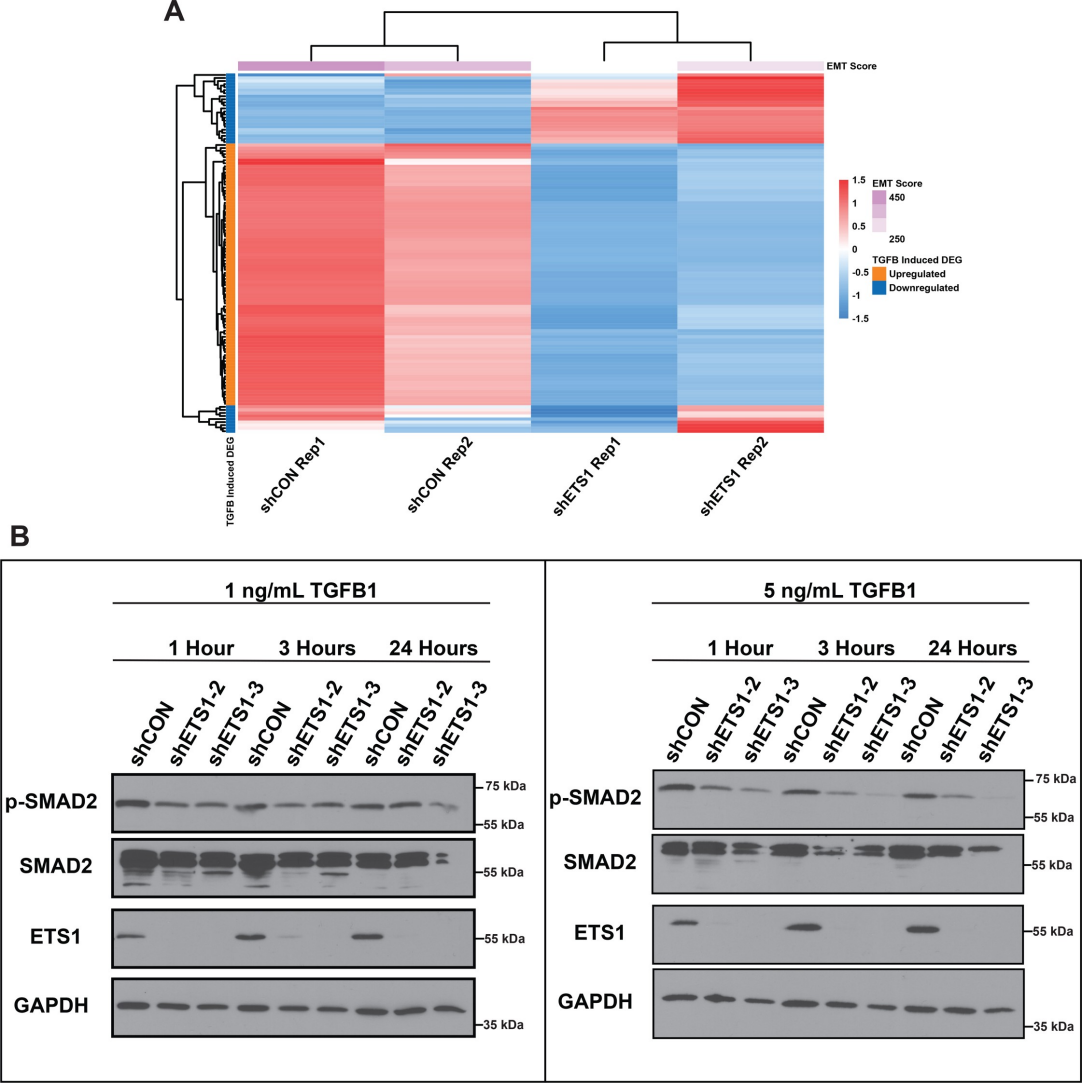
ETS1 is a key modulator of TGF-β signaling activated EMT. **(A)** Overlap between selected ETS1 target genes with the TGFβ-induced EMT signature. **(B)** Western blot showing the effect of loss of ETS1 in SCC25 cells on TGFβ signaling and activation.

Since the long-term effects of constitutive loss of ETS1 are likely to be pronounced, we followed up on a larger cohort of genes that showed differential gene expression in response to the shRNA mediated knockdown of ETS1. We found that a high percentage of the significantly altered genes (5991 out of 6727) were also direct targets of ETS1 based on ChIP-Seq data (S5 Table). To facilitate further analysis, we focused on genes that change at least 2-fold in expression, and identified 1038 such transcriptional targets of ETS1. We first used the Ingenuity Pathway Analysis (IPA) Regulator Effects tool to match significantly altered genes with their associated biological phenotypes. Interestingly, the analysis highlighted a number of predicted biological effects due to loss of ETS1, such as tumor cell migration and invasion, cell cycle progression and tumor growth, (S7 Fig). Next, we correlated the function of ETS1 to known upstream transcriptional regulators as well as the signaling cascades under their control using the IPA Upstream Regulator tool. Interestingly, we observed that the one major subset of ETS1-driven DEGs, consisting of 233 genes were significantly associated with the activation of TGF-β signaling (Fig 7). The link between TGF-β signaling and its role in inducing EMT has been established across numerous cancer models[56–58]. Our findings suggested that ETS1 could function as a driver of the mesenchymal phenotype of the SCC25 cells by acting as a regulator of TGF-β signaling. We further validated ETS1/TGF-β link by examining the enrichment of ETS1 direct targets in established gene-signatures related to TGF-β activation in cancer. We observed a significant overlap of 188 ETS1 target genes, with 118 of those displaying perfect correlation with the DEGs (Fig 8A, S6 Table, p = 4.0e-28, Fisher’s exact test) when compared with a comprehensive EMT gene signature that has been recently developed[59].

Given the likelihood that ETS1 could be driving and maintaining the mesenchymal gene expression program of SCC25 through the regulation of the TGF-β signaling pathway, we next examined the activation status of Smad2, the major receptor-activated Smad downstream of TGF-β signaling. For this purpose, the stably transfected control and the ETS1 sh2 and sh3 transduced SCC25 cells were stimulated with 1ng/ml and 5ng/ml of TGF-β1 for 1, 3 and 24 hours and examined by western blot analysis. Interestingly, we observed a dramatic decrease in overall levels of p-Smad2 in the ETS1kd cells as compared to the control (Fig 8B). Additionally, we observed a decrease in the overall phosphorylation levels of p-Smad2 over time, which was not observed in the control cells; the shCON cells maintained high levels of p-Smad2 after TGF-β1 stimulation in all durations of treatment. Similar effects of TGF-β signaling, specifically the reduced levels of p-Smad2 in the ETS1kd as compared to the control cells were also observed for SCC4 cells, particularly after 24 hours of TGF-β treatment (S8A Fig). These findings suggest ETS1 plays a role in maintaining the overall activation state of the TGF-β signaling pathway.

**Fig 9:**
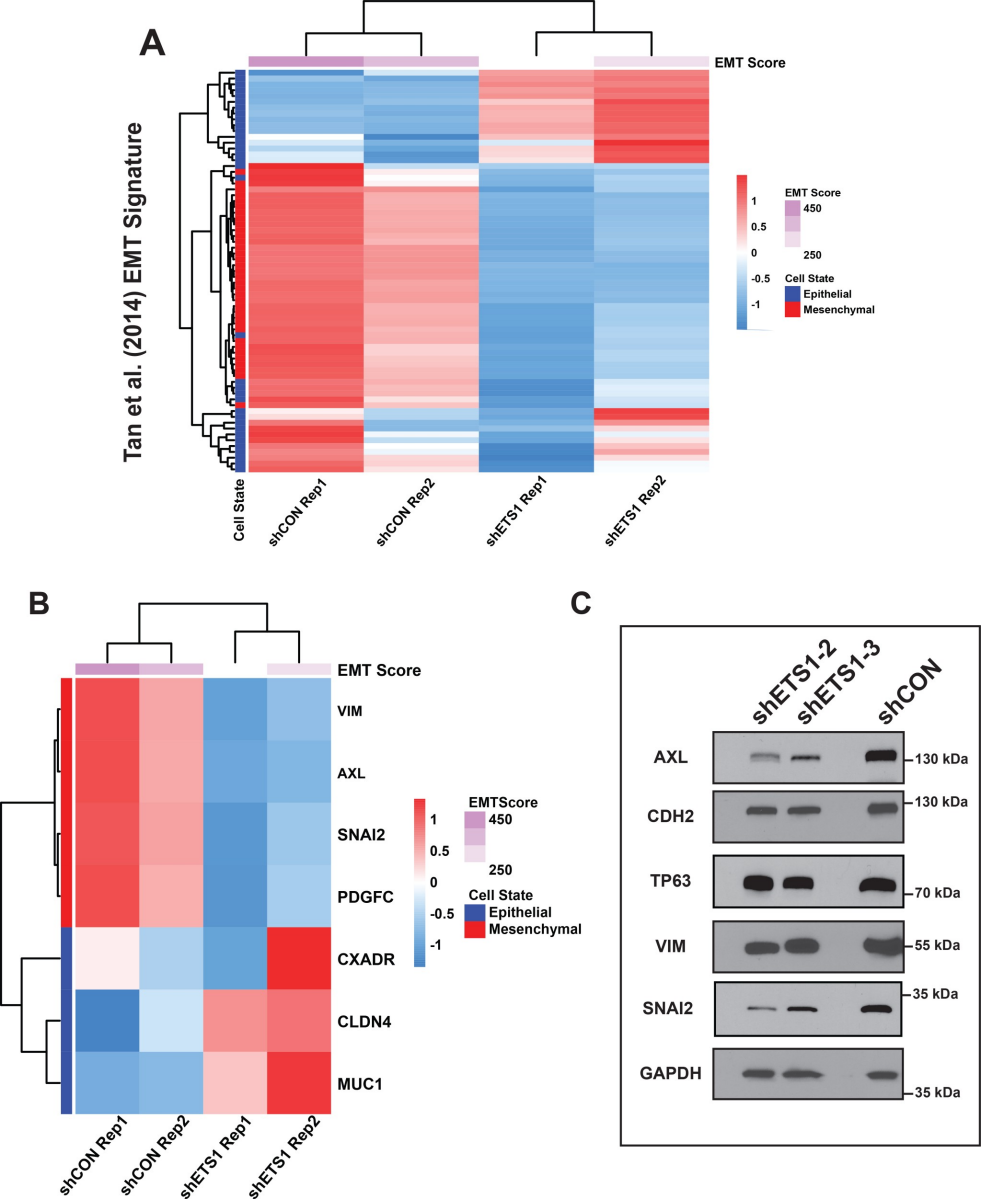
ETS1 is a key modulator of EMT. (**A)** Heat map of direct transcriptional ETS1 target genes that overlapped with published EMT Signature (Tan et al., 2014). **(B)** Heatmap showing the EMT score and the relative transcript levels of EMT and MET markers within SCC25 cells expressing shETS1 compared to shCON cells. **(C)** Western blot showing the expression of selected markers in SCC25 cells either expressing shETS1-2 or shETS1-3 as compared to cells expressing shCON. GAPDH, loading control.

The strong association of ETS1 function with TGF-β induced EMT pathway prompted us to further validate this relationship with an independent gene-signature aimed at quantifying the EMT status of a tumor. Towards this end, we utilized a EMT signature that has been derived from the common molecular EMT features of several epithelial cancer types[60] and found a significant overlap with the SCC25 ETS1 target genes (Fig 9A, S7 Table, 143/315, p = 1.49e-9 Fisher’s Exact Test). As evident from the heatmap, a number of key EMT related genes, such as VIM, AXL and SNAIL2 were downregulated after knockdown of ETS1. In contrast, genes representing the reverse state of Mesenchymal Epithelial Transition (MET) were upregulated, implying a potential repressive role for ETS1 in keeping the epithelial state of SCC25 suppressed (Fig 9B). We independently confirmed the altered expression of the protein levels of some of these EMT/MET associated genes by western blot analysis of both the SCC25 and SCC4 control and ETS1sh2 and sh3 knockdown cells (Fig 9C, S8B Fig).

### ETS1 directs a mesenchymal gene expression program in HNSCC

The strong association of ETS1 with the MS subtype of HNSCC tumors prompted us to compare the mesenchymal specific gene signature derived from the TCGA RNA-Seq dataset with the ETS1 target genes in SCC25. There was a significant overlap between the two sets of gene lists (p = 1.84e-5, Fisher’s Exact Test) resulting in the identification of a core ETS1 driven 169 Mesenchymal gene signature (ETS1 MGS, S8 Table). Interestingly, nearly 18% of these genes were found to associated with a SE and included AXL, a known regulator of cancer associated EMT[61]. Spurred by these findings, we decided to test whether the ETS1 MGS could act as an independent means of classifying HNSCC, especially as the core ETS1 MGS did not include any of the classical EMT markers. After classifying each tumor as EMT-High, EMT-Mid and EMT-Low, based on the distribution of EMT Scores, we utilized principal component analysis (PCA) to measure and visualize the variance in gene expression within TCGA HNSCC Tumors as a function of the ETS1 MGS. The resulting PCA plot displayed a striking degree of variability between the different tumors, however; the centroids of each group of tumors appeared as clearly separated entities (S9 Fig). Indeed, the first two principal components were associated with the total expression of the ETS1 MGS, which in turn was highly correlated with the distribution of EMT scores within the tumors. This finding indicated that the ETS1 MGS was able to cluster HNSCC based upon an intrinsic Mesenchymal gene expression program.

As a final test of a possible functional role of ETS1 as a regulator of EMT in tumorigenesis, we probed the scRNA-Seq datasets of HNSCC that have been described recently [28]. In this study, a sub-population of cells were identified that were defined by a partial-EMT (p-EMT) gene-expression program, and enrichment for TGF-β signaling. To identify a possible association of ETS1 with the p-EMT signature, we examined the enriched genes identified in these cell populations to see if they are also represented in the ETS1 MGS. Interestingly, we observed a significant overlap between the ETS1 MS Signature derived from our study and the top 100 genes enriched in p-EMT cells (p-value = 7.144e-12, hypergeometric test) as well as a significant overlap with the non-negative factorization analysis derived p-EMT gene signatures (p = 2.514e-21, hypergeometric test) (S10 Fig). Taken together these findings are highly suggestive of a potential role of ETS1 in maintaining the EMT state in HNSCC, possibly as a master regulator of the TGF-β signaling pathway.

## Discussion

The complex heterogeneity of HNSCC, similar to cancers from other anatomical locations, is clearly evident in the distinct gene expression profile of its subtypes. To better understand this heterogeneity, it is important to dissect the complex molecular circuitry that governs the dysregulated transcriptional networks in a given subtype. One powerful approach towards this end is to identify and characterize the master TFs which are likely to drive, maintain and elicit the tell-tale characteristics of a given tumor subtype. Among such oncogenic TFs, the ETS family of proteins are emerging as crucial mediators of tumorigenesis. Indeed, ETS factors are aberrantly activated in cancer by a slew of molecular mechanisms that include chromosomal translocation, overexpression, or post-translational modifications[62]. These ETS driven alterations in turn induce dysregulated gene expression programs that are likely to be essential in various facets of tumorigenesis.

As a prototypic and founding member of the ETS family, ETS1 has been widely studied and implicated in a variety of solid tumors[63]. Overall, data from such studies have often linked ETS1 expression to advanced state of epithelial cancers with poorer differentiation, higher invasive activity and angiogenesis, an increased risk to metastasis and a higher tendency to acquire drug resistance–all hallmark features of cancer. Despite such clear associations, the molecular attributes of ETS1, in particular its target gene repertoire and function in context of a defined tumor subtype remains ill-understood. Here we have comprehensively examined ETS1 in HNSCC, where we find ETS1 to be specifically enriched in expression across cell lines, human tumors and PDXs representing the mesenchymal subtype and associated with poor tumor outcome. To gain mechanistic insights into its function, we have undertaken genomic and epigenomic based identification and analysis of the ETS1-dependent cistrome in the SCC25 cell line, which has revealed several interesting findings.

Foremost, we uncovered that the direct and indirect target genes of ETS1 are quite extensive and impinge upon virtually all key aspects of tumorigenesis. Our findings are in some ways different from similar studies performed in prostate cancer cells, [53] where the number of ETS1 bound elements are significantly smaller compared to those in the SCC25 cells. We suspect that this might be in part due to the robust nature of our ChIP experiments, having utilized two independent antibodies. Our ETS1-genomic interactions are further strengthened by the overlay of active histone marks, which has allowed us to gain exciting insights into the ETS1-chromatin interactions and the distinct clusters of regulatory elements that are bound by ETS1. Importantly, the proclivity of ETS1 to be enriched for binding in the Super-Enhancer regions and for itself to be regulated by a SCC25 Super-Enhancer is broadly suggestive of its crucial function and specifically the existence of a potential feed-forward loop. It is worth noting that similar enrichment of ETS motifs and the binding of ETS2 have been reported in Super-Enhancers identified in other contexts, such as skin SCC and nasopharyngeal cancer[64, 65]. The remarkable structural and functional similarity of ETS1 and ETS2 hints at an interesting crosstalk and possible redundant interplay between these closely related TFs in HNSCC subtypes. Recent studies however suggest that ETS1 and ETS2 can also direct gene expression programs in opposite directions allowing these TFs to switch between oncogenic and tumor suppressive functions in cell-type specific manner [66]–whether similar mechanisms are at play in HNSCC begs further investigation.

We have performed extensive examination of the ETS1 target genes by integrating RNA-Seq and ChIP-Seq experiments. These studies have reaffirmed the notion that ETS1 casts a wide net in regulating the transcriptional network of cancer cells, affecting virtually all key hallmarks of cancer. Prominent among these ETS1-dependent processes and pathways are cell proliferation and migration, angiogenesis, inflammation and in keeping with the mesenchymal nature of the SCC25 cells, EMT. Interestingly, many of these processes are also affected in our previously described transgenic mouse model of ETS1 overexpression in squamous epithelium that leads to dysplasia and inflammation[67, 68]. In this context, it is also worth noting that although ETS1 has not firmly secured its place alongside the classical EMT-TFs, prevailing evidence from past literature and our present study illustrate its growing and pervasive role in Type I (developmental), Type II (inflammation and wounding) and Type III (neoplasia) EMT[63]. Indeed, a recent pan cancer survey of EMT markers across the TCGA has revealed ETS1 to be one of the highest ranked TF which showed significant EMT correlations within multiple tumor types that span various histological or cellular backgrounds[69].

We posit that our findings on the ETS1-TGF-β-EMT axis in SCC25 and SCC4 cells are considerably strengthened by similar trends in cells with partial-EMT that are localized to the leading edge of primary HNSCC tumors[28]. These associations might have implications for a range of other solid tumors, some of which are also endowed with mesenchymal features, such as Claudin-low breast cancer. Our findings are also supported by results from studies on CD90(+) tumor initiating cell population from esophageal squamous cell carcinoma, where the deregulation of an ETS1/MMP signaling pathway and EMT figure prominently[70]. Taken together our discovery of ETS1 as a biomarker of mesenchymal HNSCC and a master regulator of key EMT genes offers promising new avenues for targeted therapeutic possibilities that are tumor subtype specific. This includes possible re-purposing of clinically approved drugs such as Dasatinib, which has been shown to target ETS1 for proteasomal degradation[51].

## Methods

### Cell culture studies

SCC25 and SCC47 cell lines were purchased from ATCC and MilliporeSigma, respectively. Cell lines UMSCC29, UMSCC23 and UMSCC103 were obtained from Dr. Thomas Carey (University of Michigan). HSC-3 and CAL-27 cell lines were generously donated by Dr. Manish Bais (Boston University). SCC4 cell line was obtained from Dr. James Rheinwald (Harvard University). SCC25 and SCC4 was maintained in DMEM/F12 high glutamine culture media supplemented with 10% fetal bovine serum (FBS), 400 ng/mL hydrocortisone and antibiotics. All other cell lines were maintained in high glutamine DMEM containing 10% FBS, 1% GlutaMax, 1% Non-Essential Amino Acids, and antibiotics. The identities of all cell lines were confirmed via STR profiling.

### Knockdown of ETS1

For siRNA mediated knockdown of ETS1, siGENOME siRNA, which consists of 4 pooled siRNAs, were used. Biological replicates of SCC25 cells were treated with either 75 pmol of non-targeting siRNAs or siRNAs targeting ETS1, using Lipofectamine RNAiMAX. Lentiviral transduction of SCC25 and SCC4 cells expressing either a non-targeting shRNA or ETS1-targeting shRNAs (shETS1-2, shETS1-3) was achieved by the One-Shot LentiX Kit (Clontech) followed by puromycin selection (2 μg/mL).

### Western blot analysis

Cells were grown to ~90% confluency and proteins were extracted via addition of Laemmli Sample Buffer directly to the dish. Western blot analysis was performed using a standard protocol as described before(68). The antibodies used were: (C4, Santa Cruz Biotechnology), p-Smad2 (GTX111075, GeneTex), Smad2 (GTX111075, GeneTex), Vimentin (Epitomics), Snai2 (Santa Cruz Biotechnology), ETS2 (GTX104527, GeneTex), N-Cadherin/CDH2 (BD Biosciences), TP63 (4A4, Santa Cruz Biotechnology), AXL (Cell Signaling Technologies). The MAB374 antibody (EMD Millipore) was used to detected GAPDH as a loading control at 1:60,000 dilution.

### MTT assay

The control shRNA and ETS1 shRNA transduced SCC25 and SCC4 cells were seeded into a 96-well plate (10^4^ cells/well) and incubated for 24, 48, 72 and 120 hours, and then were subjected to the Vybrant MTT Cell Proliferation Assay Kit (Thermo Fisher) according to the manufacturer’s instructions. The viability of the cells was assessed by measuring the absorbance at 492 nm using a Cytation 5 Cell Imaging Multi-Mode Reader (BioTek Instruments). All experiments were performed in triplicate. The cell proliferation differences were reported in percent change in growth, which measures the changes in cell number relative to the starting 24-hour time point.

### Wound healing assay

Both control and ETS1 knockdown of SCC25 and SCC4 cell lines were seeded into 6-well plates and grown until confluence. Cells were treated with 10 μg/mL of mitomycin C (Enzo Life Sciences) for 2 hours prior to the initiation of the wounding. A scratch was then made across the center of each well using a sterile pipette tip, and non-adherent cells were washed off with Phosphate-Buffered Saline. Cells were grown in regular media and images of the wound were taken immediately and after in 12–24 hour intervals until wounds were completely closed. All experiments were performed in triplicate and the data is presented as percent change in area relative to time 0.

### Transwell assay

The Corning Matrigel Invasion Assay (Corning BioCoat Matrigel Invasion Chambers, 8 μm, Corning) was used to characterize the ability of SCC25 cells to invade through a 3D matrix. Briefly, inserts were thawed at room temperature and reconstituted using serum-free DMEM/F12 media. SCC25 cells with non-targeting control or ETS1 targeting shRNA, were added to quadruplicate inserts at a concentration of 5x10^5^ cells/mL in serum free media. Serum containing media was placed in the well beneath the insert and the entire insert-containing 24 well plate was allowed to incubate at 37°C at 5% CO_2_ for 24 hours. After incubation, the top of the membrane of the inserts was swabbed to remove cells that had not invaded through the matrix and membrane. The wells were then washed and aspirated and subsequently fixed in 100% Methanol for 10 minutes at -20°C. Methanol was removed and inserts were washed 3 times and subsequently stained using a DAPI stain (Cell Biolabs). The insert membranes were placed on glass slides and visualized using the Cytation 5 Cell Imaging Multi-Mode Reader using the 405nm excitation laser and using the DAPI filter. Stained nuclei were counted using the supplied software in five separate fields of each insert.

### TGF-β1 Treatment of SCC25 and SCC4 Cells

SCC25 and SCC4 cells stably expressing a non-targeting shRNA or ETS1 targeting shRNA, were plated into 6 well plates at 250,000 cells per well in duplicates. The cells were allowed to adhere to the plates for 12 hours and were subsequently serum starved for 24 hours. After serum starvation, the cells were either treated with a solution of 1ng/mL, 2ng/mL or 5 ng/mL TGF-β1 (R&D Systems) for a duration of 1, 3, or 24 hours. Post-treatment, whole cell protein lysates were collected and resuspended in Laemmli Sample Buffer (Bio-Rad) for western blot analysis.

### Chromatin Immunoprecipitation (ChIP) of ETS1

The High Sensitivity ChIP-IT Kit from Active-Motif and the associated protocol was used to perform ChIP. SCC25 cells were grown to ~90% confluency and cross-linked in the supplied fixation Buffer supplemented with 0.5% Formaldehyde for 10 minutes. Lysates from the fixed cells were subsequently sonicated with a Diagenode Bioruptor to obtain sheared chromatin with an approximate fragment length of 150–400 bp. The ChIPs for ETS1 were carried out using 4 μg of the polyclonal ant-ETS1 antibodies, C20x (Santa-Cruz) and A300-501A Antibody (Bethyl Laboratories). After cross-link reversal, proteinase-K/RNAase-A treatment and DNA-purification, libraries were prepared using the ThruPLEX DNA-Seq Kit (Rubicon Genomics). ChIP DNA and input controls were then subjected to 50 bp single-end sequencing on an Illumina HiSeq 2500, which resulted in 15–25 million reads per sample.

### Head and neck squamous cell carcinoma dataset analysis

Microarray profiling of patient samples was obtained from GEO (GSE45153, GSE6791, and GSE41613). Each dataset was processed according to the protocol defined by the original study. The Cancer Genome Atlas (TCGA) RNA-seq expression datasets was downloaded from GEO (GSE62944). Briefly, featureCounts (*Rsubread* v1.14.2, [71]) generated gene-level outputs from the entire TCGA RNA-Seq repository was utilized for downstream analysis. HNSCC samples (459 tumor and 44 normal) were extracted and normalized using the median-ration method (DESeq2 v1.12.3, [72]) and subsequently transformed to Transcript per Million (TPM) values[73]. Tumors were classified into intrinsic molecular subtypes as previously described[36]. EMT scores was calculated according to the formula defined by Salt et al., 2014.

### Identification of subtype specific genes within the TCGA RNA-Seq dataset

TPM tables were generated for each tumor within the TCGA HNSC RNA-Seq dataset and subsequently used as input for classification into one of four intrinsic subtypes as previously described [36]. FeatureCounts generated gene-level counts tables for each tumor was used for DESeq2 analysis whereby tumors of identical subtype classification were treated as replicates. All possible combinations of contrast was performed and a gene was considered subtype-specific if it was commonly over-expressed across each of the subtype-subtype comparisons.

### RNA isolation and RNA-Sequencing analysis

Total RNA from HNSCC cell lines was extracted using the Direct-zol RNA MiniPrep kit (Zymo Research). For each RNA sample, cDNA libraries were prepared using the TrueSeq RNA Sample Preparation Kit (Illumina), which were then 50 bp single-end sequenced on an Illumina HiSeq 2500. Quality control metrics were performed on raw sequencing reads using the FASTQC v0.4.3 application. Reads were mapped to the Homo Sapiens genome (GRCH37 build) with TopHat2 v2.1.1, using Bowtie2 v2.2.6 as the underlying aligner [74, 75]. Reads aligning to the Ensembl GRCH37 build were quantified with featureCounts. Relative transcript abundances per experiment were reported in Transcript per million (TPM) values and were calculated from the featureCounts output generated by Rahman et al. [76]. All differential gene expression analysis was carried out using DESeq2, using an FDR cutoff of 0.1. Only protein coding genes with expression level of at least 1 TPM in a given replicate for each sample, were considered for DESeq2 analysis.

### Survival analysis of HNSCC patients

Overall survival analysis of patients in the FHCRC dataset (n = 97, Lohavanichbutr et al., 2013) was carried out using the survival R package (v2.41–3) and Kaplan-Meier plots were generated using ggplot2 v2.2.1. Patient tumor samples were deemed to have high ETS1 expression based on the median expression values. The log-rank statistic was used to calculate statistical significance between different patient classifications and their relationship to clinical outcome.

### ChIP-Seq analysis pipeline

The raw ChIP-Sequencing reads from all experiments were mapped to the Homo sapiens genome (hg19 build) using Bowtie v1.1.1 with the parameter, *m*  =  1, which removes all reads mapping to multiple genomic loci. All ChIP-Seq experiments were underwent strand cross-correlation analysis using SPP v1.10.1 and deemed acceptable if the QualityTag score was 1 or higher[77]. All identified peaks were matched to the nearest gene using GREAT analysis using default settings[78].

#### Determining ETS1 targets using IDR

To identify high-confidence ETS1 binding loci, the IDR (Irreproducible Discovery Rate) analysis pipeline (https://sites.google.com/site/anshulkundaje/projects/idr), was used to identify peaks shared across the three replicate experiments as described previously[79]. Briefly, to check for self-consistency, each bam file was randomly split into two parts with approximately equal number of reads to create pseudo-replicates. Then pooled data files were generated from all of the actual replicates by using the merge function in SAMtools v1.3. MACS2 v2.1.0 peak-calling software was used to call peaks from all three types of files (original replicates, pseudo-replicates and pooled files) under low statistical stringency (P-value: 5e-2) using a merged input as control[80–82]. The resulting MACS2 peak files were sorted by P-value and filtered to ascertain the top 100,000 peaks, which were then used as input for the IDR analysis. Based on the optimal cutoff recommended by IDR, 26970 consensus ETS1 binding sites were identified in SCC25. To visualize the correlation between ETS1 ChIP-Seq experiments, a heatmap was generated that displayed the ETS1 ChIP signal at each peak in each individual experiment, determined at a 3kb window and displayed in descending order as a heatmap using the deepTools package. The resulting ETS1 ChIP signal matrix was subjected to hierarchical clustering (Euclidean Distance, Complete Linkage).

### MEME de novo motif analysis of ETS1 ChIP-Seq

The MEME tool was used to generate a de-Novo motif from the ETS1 ChIP-Seq experiments [83, 84]. A 600 bp window was selected around each ETS1 peak summit to use as an input for the program. The selected MEME-generate motif was subjected to TOMTOM motif comparison analysis, which showed that the motif derived from genomic segments bound by ETS1 as ascertained from ChIP-seq data was most similar to the consensus ETS core motif (43). The comparison with the ETS1 motif is highlighted.

### Genomic feature assignment

The CEAS tools was used to annotate ETS1 ChIP-Seq peaks to the nearest genomic of feature of the hg19 genome assembly[85]. The promoter region was considered up to 500 bp away from a TSS and the proximal enhancer was considered 500 bp-1500 bp away. Any binding within a gene was considered intragenic, whereas any binding site greater than 1500 bp upstream or downstream was considered distal intergenic.

### Histone modification enrichment at ETS1 binding sites

Heatmap showing the ChIP-Seq signal of the H3K27Ac, H3K27Me3, H3K4Me1, and H3K4Me3 Histone modifications around a 2-kb window centered at each ETS1 ChIP-Seq peak summit. The resulting histone signal enrichment was subjected to k-Means clustering (k = 4). The fluff python package was used to generate the heatmap, using default parameters[86]. Detailed experimental methods of the epigenomic studies in the SCC25 are described in a separate manuscript currently under review (Tsompana et al.,).

### Determination of Enhancers and Super-Enhancers bases on H3K27Ac marks

Biological replicates of H3K27Ac ChIP-Sequencing data from SCC25 cells (manuscript under review (Tsompana et al,.) were aligned to the human genome as described above. Narrow-Peaks were called using MACS2 v2.1.0 using the following parameters (-p 0.01 –nomodel–extsize 150). The resulting narrowPeaks files were converted to gff format and used as inputs for the ROSE Algorithm, which was run using default parameters along with appropriate input controls to generate typical and Super-Enhancers [47, 48].

### Visualization of genomic enrichment of H3K27Ac signal

SCC25 H3K27Ac ChIP-Seq replicate experiments were merged. The deeptools program was used to convert the H3K27Ac signal to RPKMs (Reads per Kilobase per Million Mapped Reads) and was subsequently summed across the genome within 10 base-pair bins at the selected loci.

### Motif enrichment analysis of Super-Enhancers

To determine the top enriched DNA-Binding Motifs of transcription factors found within the Nucleosome Free Regions (NFR) of SCC25 Super-Enhancers, first NFRs were determined using the Homer findPeaks tool with the–nfr flag turned on. The AME tool was used to determine enriched motifs found within the JASPAR Vertebrates 2014 database. Motifs were ranked according to adjusted p-value.

### Gene ontology/pathway enrichment analysis

For ETS1 ChIP-Seq data, GREAT tool[87] was used to annotate binding loci to the nearest gene as well to carry out gene-set enrichment analysis using default parameters. For RNA-Seq data, the MSigDB was used to identify gene-sets with significant overlaps with the various gene signatures derived above using the hypergeometric test as well as controlling for multiple testing using the Benjamini-Hochberg correction (cutoff = 0.05).

### Network analysis

To visualize the ETS1 driven molecular signature and its relationship to possible biological phenotypes, Ingenuity Pathway analysis (IPA, Qiagen) was used. A causal network, which construct relationships between the changes in gene expression after knockdown of ETS1 and the downstream phenotypic and molecular changes occurring in SCC25, was created using the Regulator Effect tool under default parameters.

### Principal component analysis of the TCGA HNSC dataset using the ETS1 mesenchymal signature

The expression of the ETS1 Mesenchymal Signature within the TCGA HNSC Dataset was used as input for PCA analysis. Each tumor’s EMT score was calculated and Fisher-Transformed. Tumors were classified as EMT High, Neutral or Low, based on the distribution of EMT scores, with Low being defined in the first quartile, High as the third and Neutral as in between.

## Supporting information

S1 Fig Enriched biological processes associated with the subtypes of HNSCC Tumors in the TCGA dataset. Bargraphs displays the top enriched biological processes (GO Consortium) within sets of Atypical (A), Basal (B), or Classical (C) subtype-enriched genes as determined by DESeq2 analysis. (D) Boxplot displaying the HNSCC subtype-specific distribution of ETS2 expression.
(PDF)

S2 Fig ETS1 Expression is highly correlated with the expression of classical EMT markers. **(A)** Heatmap showing the relationship between ETS1 and the constituents of the EMT score within the TCGA HNSC RNA-Seq dataset. Tumors are displayed on each row and ranked in ascending order based on TPM score. **(B)** Heatmap displaying the correlation of protein expression of ETS1 with the available data for the other constituents of the EMT score present in the HNSCC RPPA datasets. The EMT score was calculated based on the normalized RPPA data and each tumor was ranked in ascending order based on the tumor-specific EMT score. **(C)** Heatmap displaying the expression of the constituents of the EMT Score for the 8 HNSCC cell lines. Expression values were standardized and ranked in ascending order via the EMT Score calculated for each cell. SCC25 shows the highest overall EMT score.
(PDF)

S3 Fig ETS1 is preferentially expressed in mesenchymal cell lines that form Distinct gene expression clusters. Heatmaps showing the cross-correlation values of the top 1500 most variably expressed genes within **(A)** the Cohort of HNSCC Cell Lines (Martin et al, 2014) and **(B)** the Cancer Cell Line Encyclopedia dataset. The correlation matrix was reorganized via hierarchical clustering (Pearson Correlation, Complete Linkage). Displayed above each color is the subtype classification of each cell, EMT score and ETS1 expression level.
(PDF)

S4 Fig Genomic view of H3K27Ac signal at selected Super-Enhancers in SCC25. Visualization of the H3K27Ac signal at Super-Enhancers proximal to selected genes. Genes associated with Mesenchymal and Epithelial biological processes were highlighted.
(PDF)

S5 Fig Loss of ETS1 inhibits the ability of SCC25 cells to Invade 3D matrices. **(A)** Boxplot showing the invasive capabilities of SCC25-shETS1-3 and SCC25-shCON cells as assessed using a Matrigel Coated Transwell Assay. **(B and C)** Representative images showing the number of cells that have migrated to the bottom of the insert in either (B) shCON or (C) shETS1 SCC25 cells.
(PDF)

S6 Fig Loss of ETS1 inhibits cell proliferation and migratory properties of SCC4 cells. **(A)** Line plot displaying the differences in cellular proliferation between SCC4-shETS1 and SCC4-shCON cells, as determined by the MTT assay, (p = 0.0013, 48 Hours, T-Test and p = 0.031, T-Test, 72 Hours). **(B)** Wound scratch-healing assays for assessing cell migration. Percent area closure of the initial wound area of the SCC4-shETS1 and SCC4-shCON cells is shown in the left panel. Representative images of the wound area after 0, 24 and 48 hours are displayed in the right, wound (p = 4.321e-4, ANOVA, Tukey Post-Hoc). White hash marks denote the boundary of the wound.
(PDF)

S7 Fig ETS1 regulates numerous oncogenic phenotypes in SCC25 Cells. Direct transcriptional target genes of ETS1 were analyzed via the IPA Regulator Effects tool and displayed as networks (Organic Layout), with Nodes representing the predicted biological effects of the loss of ETS1 in SCC25 cells edges connecting differentially expressed genes that are associated with the expected phenotype. The top panel (A) represents a global view of the function of ETS1 direct transcriptional targets, whereas (B) and (C) represent selected processes.
(PDF)

S8 Fig Loss of ETS1 modulates TGF-β signaling and regulates EMT marker expression in SCC4 cells. **(A)** Western blot showing the effect of loss of ETS1 in SCC4 cells on TGFβ signaling and activation. **(B)** Western blot showing the expression of selected markers in SCC4 cells either expressing shETS1-2 or shETS1-3 as compared to cells expressing shCON. GAPDH, loading control.
(PDF)

S9 Fig The ETS1 Mesenchymal gene signature organizes TCGA HNSCC tumors into distinct PCA centroids based on degree of EMT activation. PCA plot showing the variance in gene expression underlying HNSCC tumors as a function of ETS1 Mesenchymal Gene Signature. Each point is colored according to its respective Fisher-Transformed EMT Score. The centroids of each subgroup of tumors are represented as large circles with each tumor baring that classification projecting from its origin. The ellipses represent the confidence intervals (0.95) for each EMT classification.
(PDF)

S10 Fig Direct targets of ETS1 are enriched in p-EMT enriched HNSCC tumor subpopulations as determined by scRNA-Seq. Heatmaps displaying the change in expression of selected ETS1 target genes in SCC25 that were enriched in the p-EMT subpopulation of HNSCC Cells. Panel (A) represents the overlap of ETS1 targets with the top 100 genes that were enriched within the p-EMT subpopulation of HNSCC tumor cells, whereas panel (B) displays the overlap of ETS1 targets with the total number of genes specific to the p-EMT population of cells as determined via NMF (non-negative matrix factorization) analysis.
(PDF)

S1 Table Mesenchymal subtype enriched genes in the TCGA HNSCC datasets. Columns A represent the genes that were significantly enriched in the MS subtype of HNSCC as determined via DESeq2 analysis, whereby Differentially Expressed Genes (DEGs) were calculated between the MS subtype and each other subtype (AT, BA, CL, Columns B-D respectively). Only genes that were statistically significant, overexpressed specifically in MS, and shared across all MS-to-other subtype comparison were selected. Column E represents the mean log2FoldChange of the selected genes, across all comparisons. Additional sheets contain the list of enriched genes for the Atypical, Basal and Classical subtypes.
(XLSX)

S2 Table IDR generated ETS1 targets in SCC25 cells. Columns A-C represents the genomic coordinates (hg19) of the 26969 ETS1 ChIP sites in SCC25 shared across three replicate ChIP-Seq experiments as determined via the IDR (Irreproducible Discovery Rate) protocol.
(XLSX)

S3 Table List of epigenetically delineated genomic targets of ETS1. The three sheets represent the sets of ETS1 targets that were defined by their unique and proximal histone modification enrichments, as determined via FLUFF clustering analysis. Sheet 1 (cluster 1) represents genomic loci enriched for active enhancers (H3K27Ac^High^:H3K4Me1^High^), sheet 2 (cluster 2) represents active promoters (H3K27Ac^High^:H3K4Me3^High^), and sheet 3 (cluster 3) represent poised enhancers (H3K27Me3^High^:H3K4Me1^High^:H3K27Ac^Low^). Columns A-C in each respective sheet represent the genomic coordinates of each ETS1 target.
(XLSX)

S4 Table List of rose algorithm determined stitched enhancer loci in SCC25 cells. The raw output of the Rose analysis of merged SCC25 H3K27Ac ChIP-Seq replicate experiments. This represents the complete collection of identified Typical Enhancers and Super-Enhancers.
(XLSX)

S5 Table Genes dependent upon ETS1 function in SCC25 cells. The output of DESeq2 analysis of the transcriptomic profile comparisons of SCC25 cells either with ETS1 knocked down using shRNA and compared with non-targeting control shRNA (Sheet 1) or SCC25s transiently transfected with ETS1-targeting siRNA and compared with non-targeting control siRNA (Sheet 2). Only genes that were statistically significant (q-value ≤0.1) are shown. The overlap between the genes bound by ETS1 as determined by the IDR Optimal ETS1 ChIP-Seq analysis and the significantly altered genes as determined from the shRNA knockdown of ETS1 in SCC25 cells is displayed in sheet 3.
(XLSX)

S6 Table Direct targets of ETS1 associated with the TGF-B1 activated EMT signature. Column A represents the direct targets of ETS1 that overlap with the TGF-B1 Activated EMT signature. Direct targets of ETS1 were determined by the intersection of genes bound by ETS1 as determined via ChIP-Seq analysis with genes differentially expressed after loss of ETS1 expression after stable shRNA knockdown. Columns B-E represent TPM expression values of those highlighted genes within the replicates of SCC25 stably transfected with non-targeting shRNA (B-C) and with ETS1 targeting shRNA (D-E).
(XLSX)

S7 Table Direct targets of ETS1 that overlap with the Pan-Cancer EMT signature. Column A represents the direct targets of ETS1 that overlap with the Pan-Cancer EMT Signature (Tan et al., EMBO Mol. Med. 2014 Oct; 6(10): 1279–1293). Direct targets of ETS1 were determined by the intersection of genes bound by ETS1 as determined via ChIP-Seq analysis with genes differentially expressed after loss of ETS1 expression via stable shRNA knockdown. Only genes whose change in expression matched with the Pan-Cancer EMT Signature were highlighted. Columns B-E represent TPM expression values of those highlighted genes within the replicates of SCC25 stably transfected with non-targeting shRNA (B-C) and with ETS1 targeting shRNA (D-E).
(XLSX)

S8 Table ETS1 Mesenchymal gene signature. Column A represents the genes associated with the ETS1 Mesenchymal Gene Signature, as determined (details in the methods section). Columns B-G represent the raw DESeq2 generated output.
(XLSX)
