# Supplementary figures and images for: Molecular dissection of the oncogenic role of ETS1 in the mesenchymal subtypes of head and neck squamous cell carcinoma
Source: PLoS Genet. 2019 Jul 15;15(7):e1008250. doi: 10.1371/journal.pgen.1008250 (PMC6657958; doi:10.1371/journal.pgen.1008250)

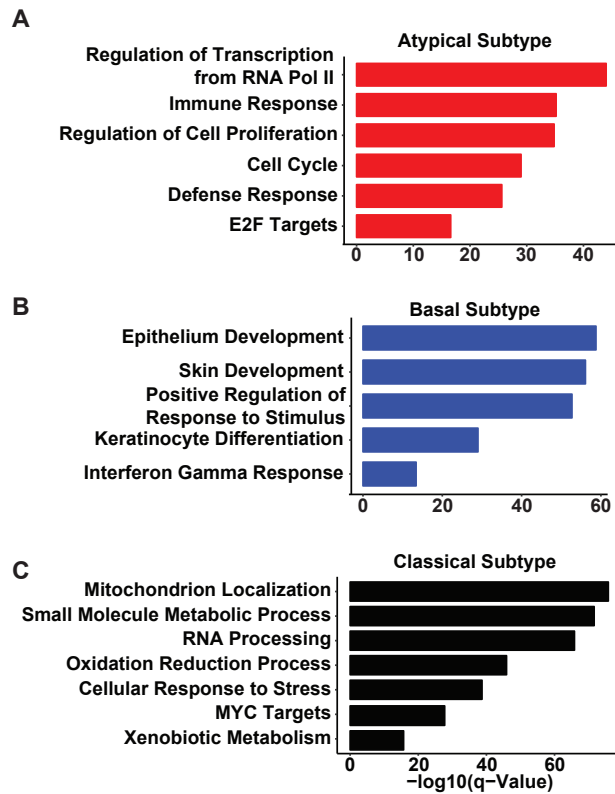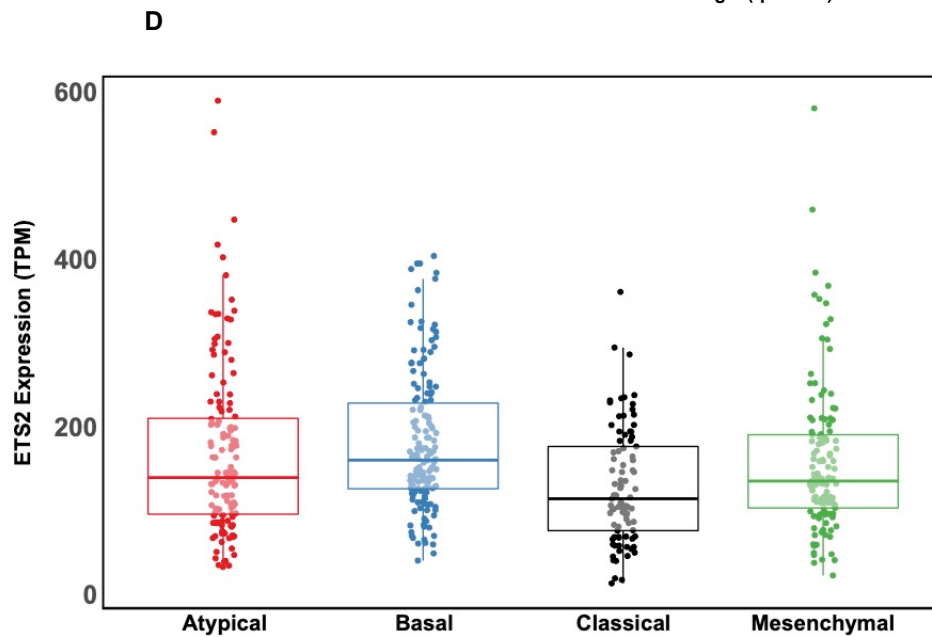

Supplement: S1 Fig — Bargraphs displays the top enriched biological processes (GO Consortium) within sets of Atypical (A), Basal (B), or Classical (C) subtype-enriched genes as determined by DESeq2 analysis. (D) Boxplot displaying the HNSCC subtype-specific distribution of ETS2 expression. (PDF) [file pgen.1008250.s001.pdf]

A

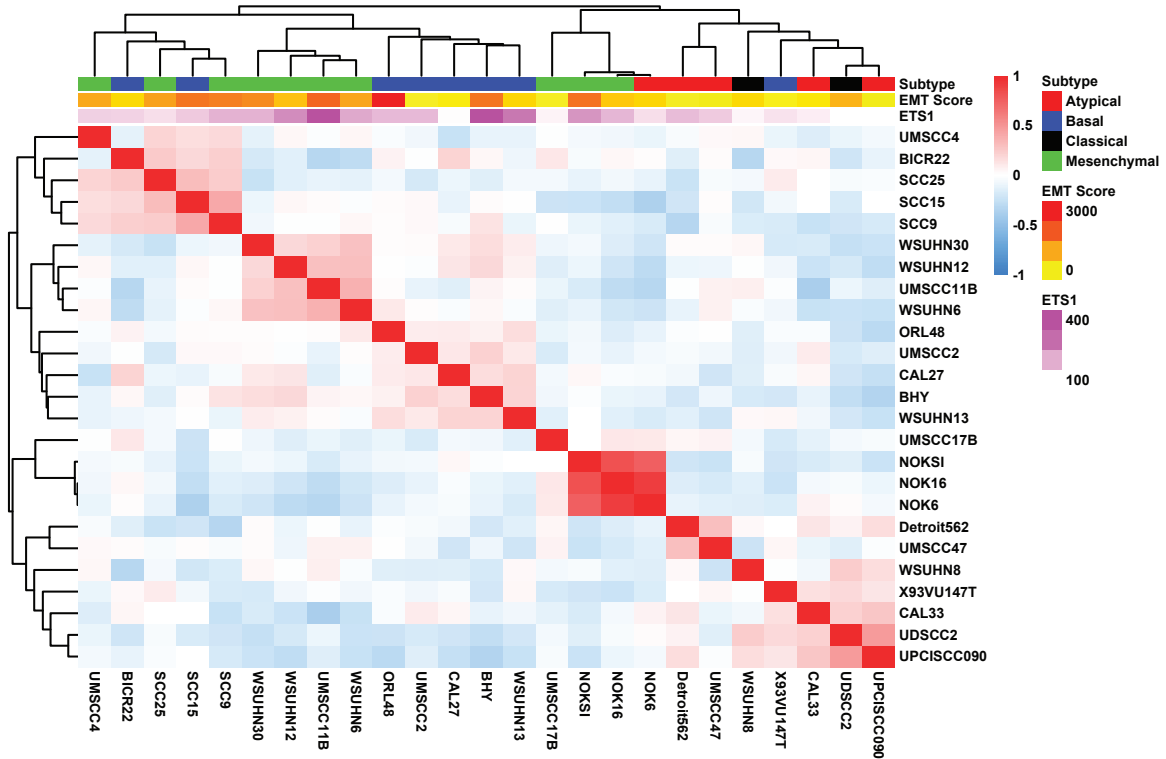

B

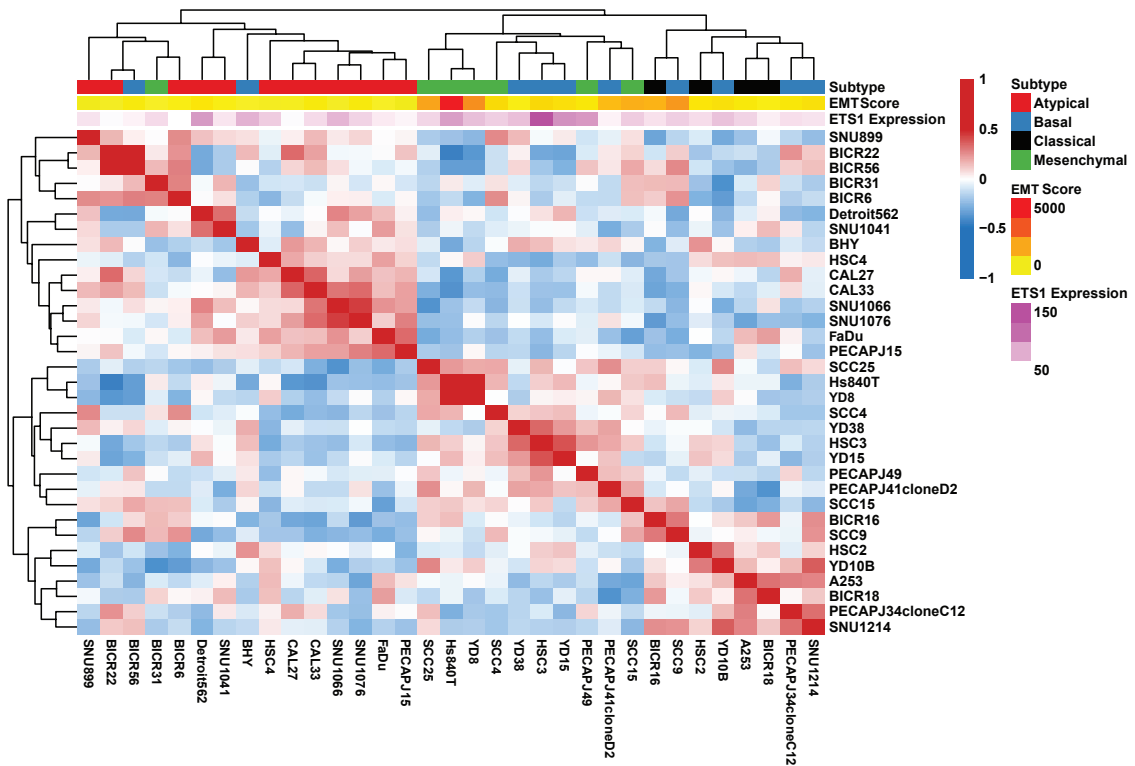

Supplement: S3 Fig — Heatmaps showing the cross-correlation values of the top 1500 most variably expressed genes within (A) the Cohort of HNSCC Cell Lines (Martin et al, 2014) and (B) the Cancer Cell Line Encyclopedia dataset. The correlation matrix was reorganized via hierarchical clustering (Pearson Correlation, Complete Linkage). Displayed above each color is the subtype classification of each cell, EMT score and ETS1 expression level. (PDF) [file pgen.1008250.s003.pdf]

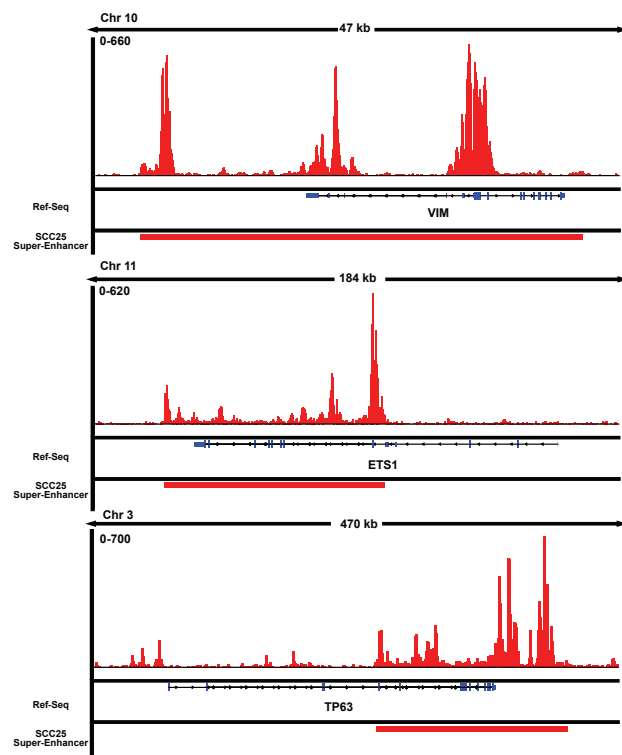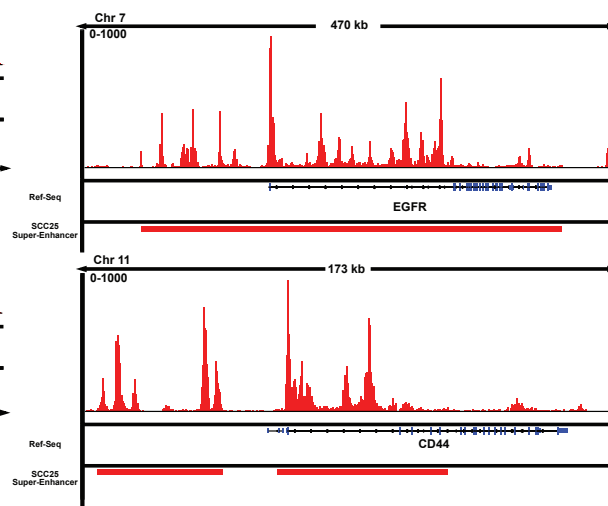

Supplement: S4 Fig — Visualization of the H3K27Ac signal at Super-Enhancers proximal to selected genes. Genes associated with Mesenchymal and Epithelial biological processes were highlighted. (PDF) [file pgen.1008250.s004.pdf]

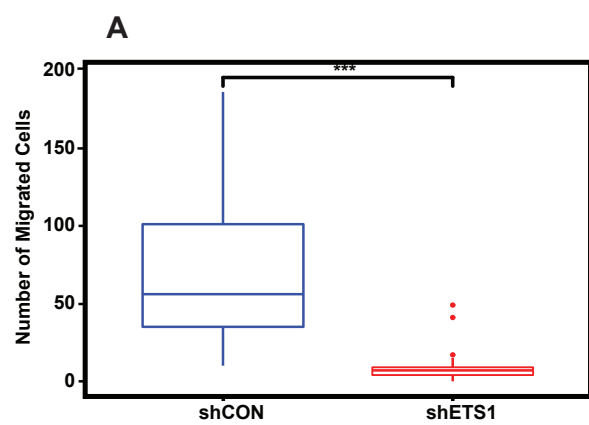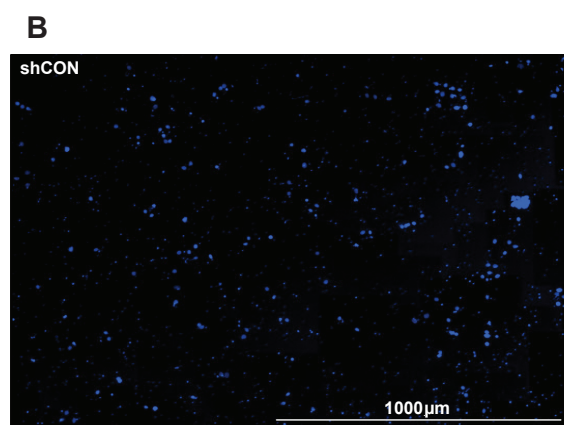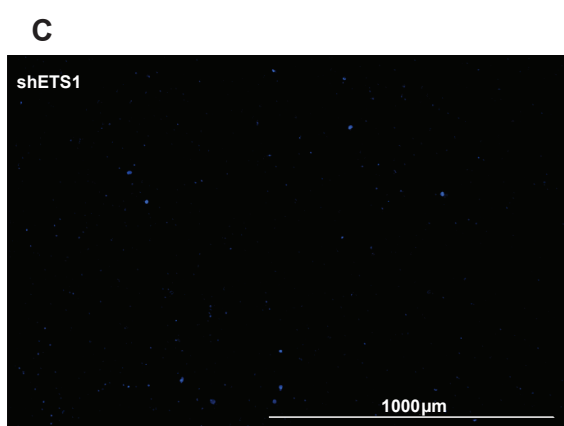

Supplement: S5 Fig — (A) Boxplot showing the invasive capabilities of SCC25-shETS1-3 and SCC25-shCON cells as assessed using a Matrigel Coated Transwell Assay. (B and C) Representative images showing the number of cells that have migrated to the bottom of the insert in either (B) shCON or (C) shETS1 SCC25 cells. (PDF) [file pgen.1008250.s005.pdf]

**A**

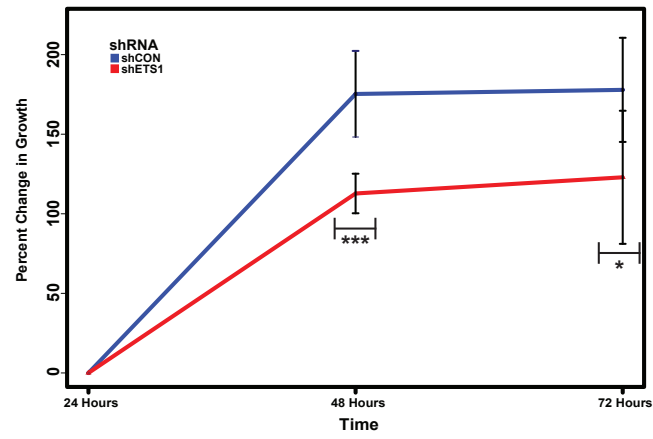

**B**

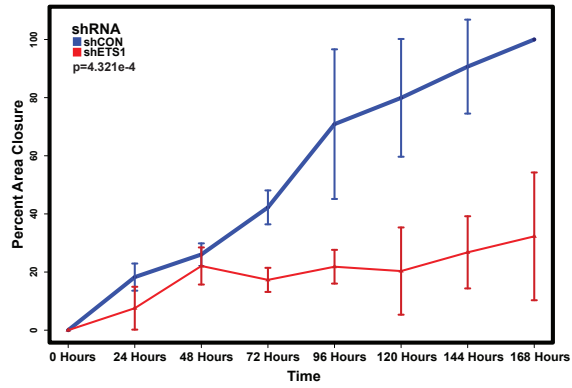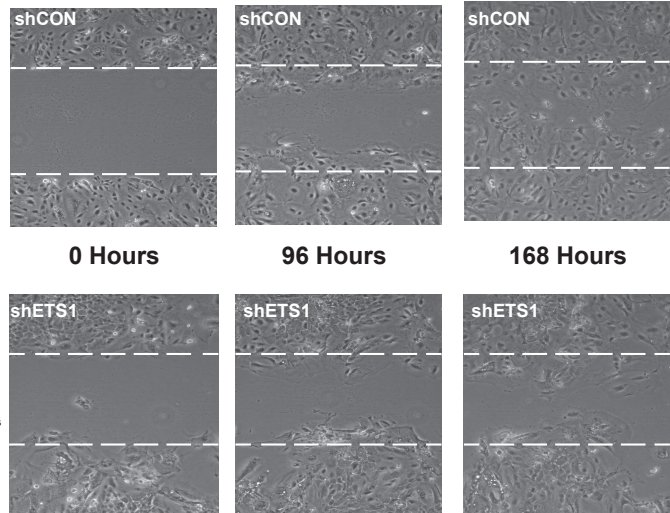

Supplement: S6 Fig — (A) Line plot displaying the differences in cellular proliferation between SCC4-shETS1 and SCC4-shCON cells, as determined by the MTT assay, (p = 0.0013, 48 Hours, T-Test and p = 0.031, T-Test, 72 Hours). (B) Wound scratch-healing assays for assessing cell migration. Percent area closure of the initial wound area of the SCC4-shETS1 and SCC4-shCON cells is shown in the left panel. Representative images of the wound area after 0, 24 and 48 hours are displayed in the right, wound (p = 4.321e-4, ANOVA, Tukey Post-Hoc). White hash marks denote the boundary of the wound. (PDF) [file pgen.1008250.s006.pdf]

**A**

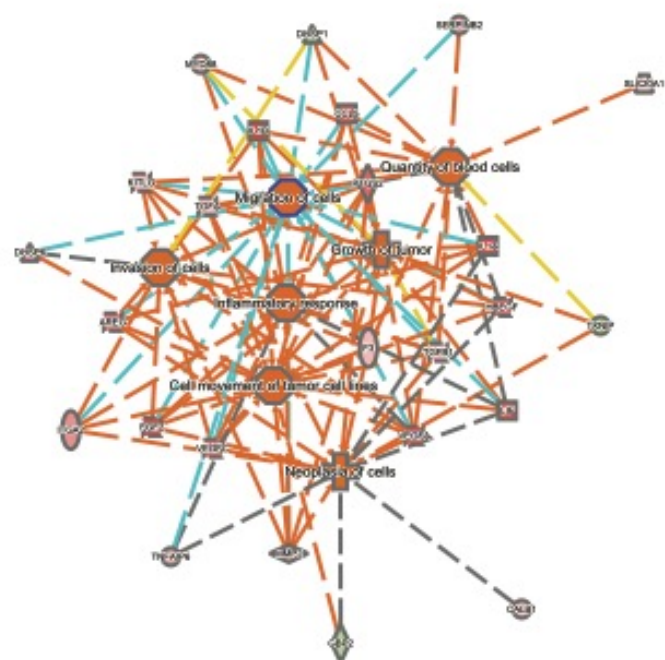

**B**

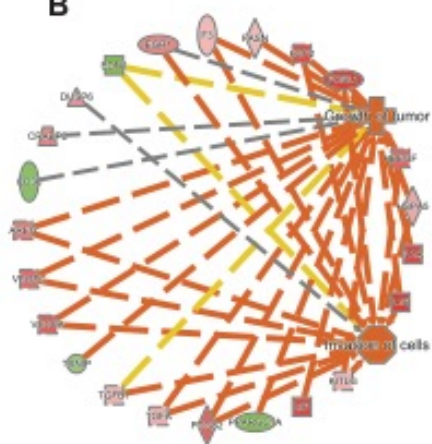

**C**

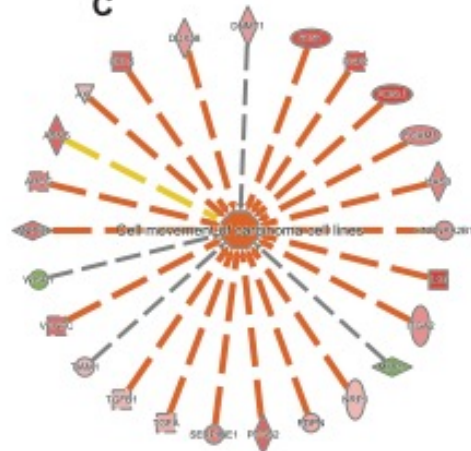

Supplement: S7 Fig — Direct transcriptional target genes of ETS1 were analyzed via the IPA Regulator Effects tool and displayed as networks (Organic Layout), with Nodes representing the predicted biological effects of the loss of ETS1 in SCC25 cells edges connecting differentially expressed genes that are associated with the expected phenotype. The top panel (A) represents a global view of the function of ETS1 direct transcriptional targets, whereas (B) and (C) represent selected processes. (PDF) [file pgen.1008250.s007.pdf]

**A**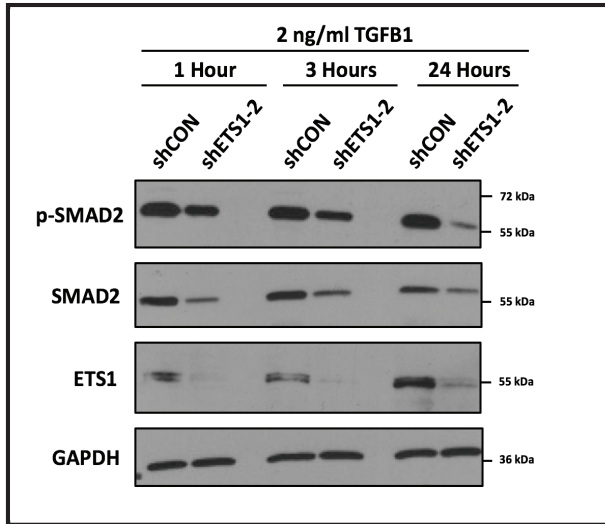**B**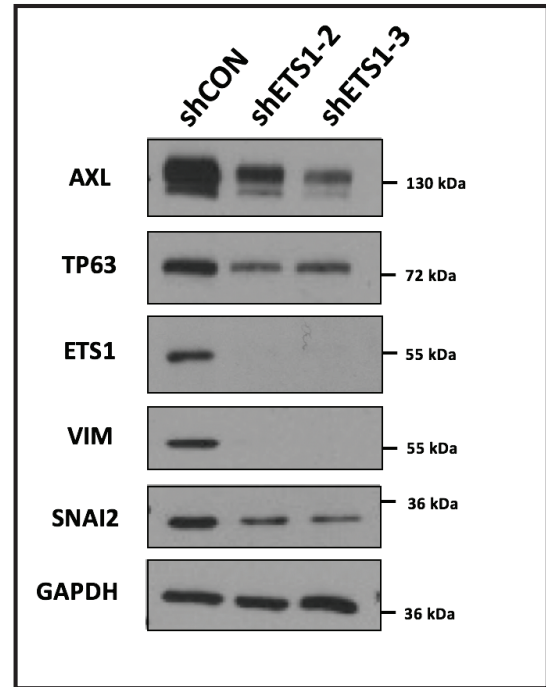

Supplement: S8 Fig — (A) Western blot showing the effect of loss of ETS1 in SCC4 cells on TGFβ signaling and activation. (B) Western blot showing the expression of selected markers in SCC4 cells either expressing shETS1-2 or shETS1-3 as compared to cells expressing shCON. GAPDH, loading control. (PDF) [file pgen.1008250.s008.pdf]

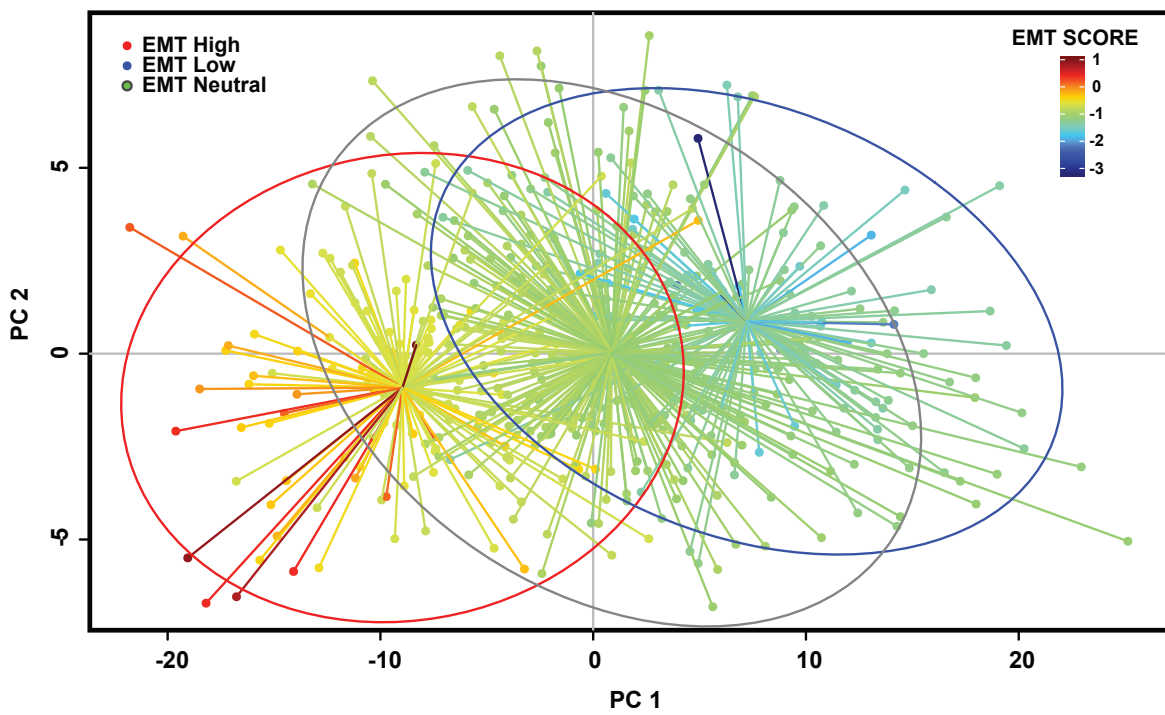

Supplement: S9 Fig — PCA plot showing the variance in gene expression underlying HNSCC tumors as a function of ETS1 Mesenchymal Gene Signature. Each point is colored according to its respective Fisher-Transformed EMT Score. The centroids of each subgroup of tumors are represented as large circles with each tumor baring that classification projecting from its origin. The ellipses represent the confidence intervals (0.95) for each EMT classification. (PDF) [file pgen.1008250.s009.pdf]

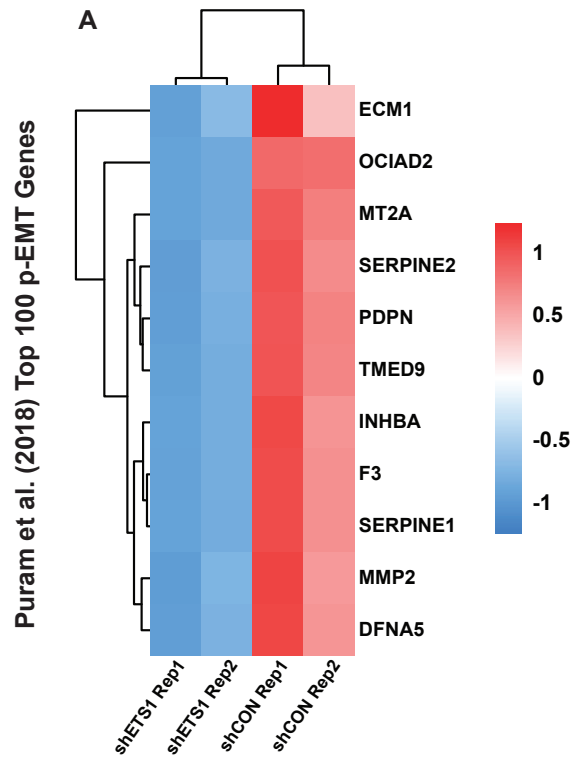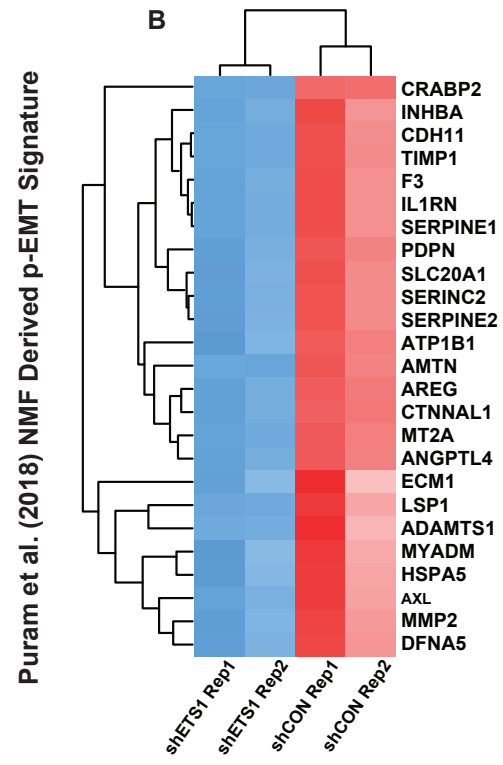

Supplement: S10 Fig — Heatmaps displaying the change in expression of selected ETS1 target genes in SCC25 that were enriched in the p-EMT subpopulation of HNSCC Cells. Panel (A) represents the overlap of ETS1 targets with the top 100 genes that were enriched within the p-EMT subpopulation of HNSCC tumor cells, whereas panel (B) displays the overlap of ETS1 targets with the total number of genes specific to the p-EMT population of cells as determined via NMF (non-negative matrix factorization) analysis. (PDF) [file pgen.1008250.s010.pdf]
