# Supplementary material for: Molecular dissection of the oncogenic role of ETS1 in the mesenchymal subtypes of head and neck squamous cell carcinoma
Source: PLoS Genet. 2019 Jul 15;15(7):e1008250. doi: 10.1371/journal.pgen.1008250 (PMC6657958; doi:10.1371/journal.pgen.1008250)
Supplement: S2 Fig — (A) Heatmap showing the relationship between ETS1 and the constituents of the EMT score within the TCGA HNSC RNA-Seq dataset. Tumors are displayed on each row and ranked in ascending order based on TPM score. (B) Heatmap displaying the correlation of protein expression of ETS1 with the available data for the other constituents of the EMT score present in the HNSCC RPPA datasets. The EMT score was calculated based on the normalized RPPA data and each tumor was ranked in ascending order based on the tumor-specific EMT score. (C) Heatmap displaying the expression of the constituents of the EMT Score for the 8 HNSCC cell lines. Expression values were standardized and ranked in ascending order via the EMT Score calculated for each cell. SCC25 shows the highest overall EMT score. (PDF) [file pgen.1008250.s002.pdf]

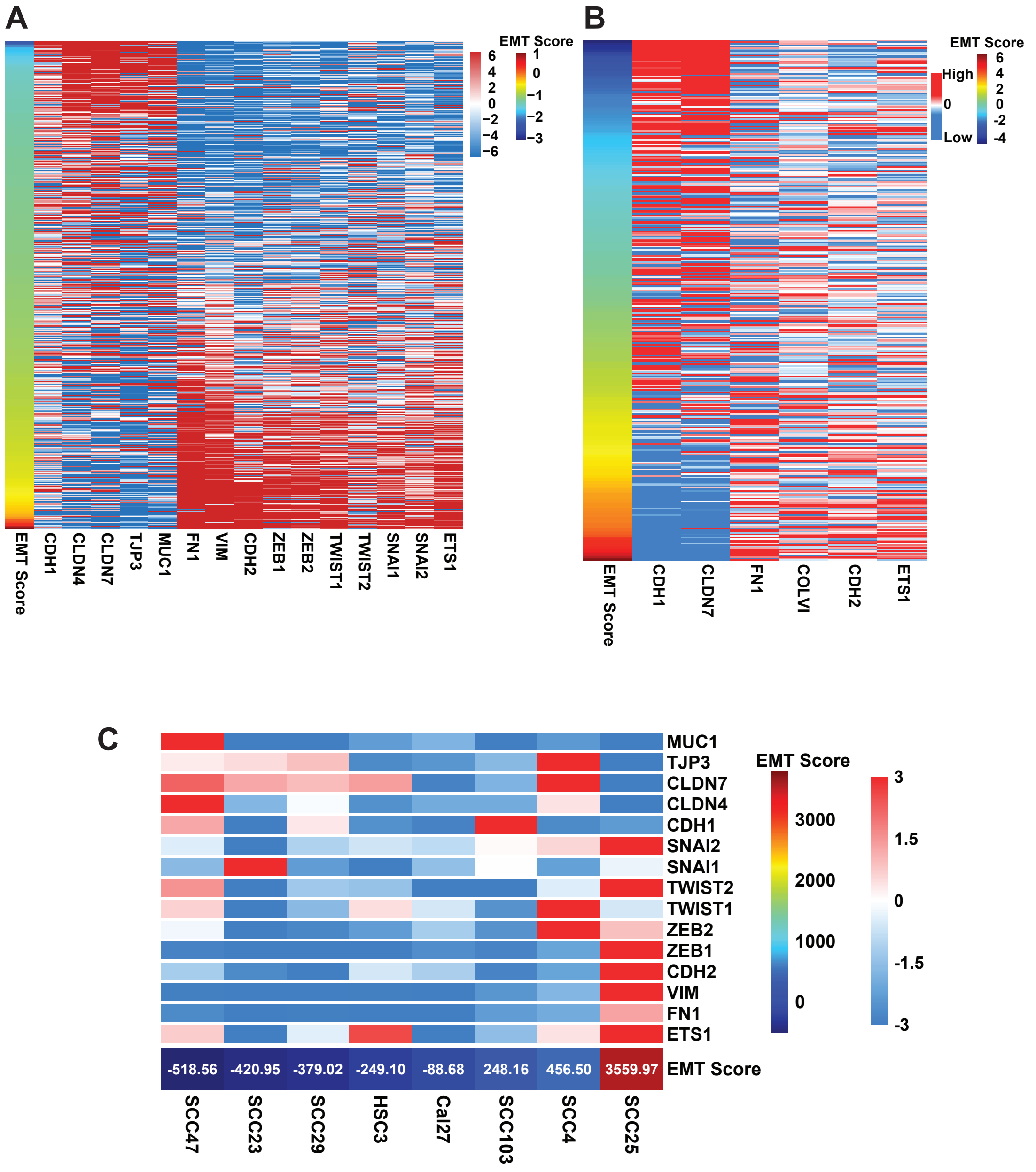
A
EMT Score
6
4
2
0
−2
−4
−6
1
0
−1
−2
−3
EMT Score
CDH1
CLDN4
CLDN7
TJP3
MUC1
FN1
VIM
CDH2
ZEB1
ZEB2
TWIST1
TWIST2
SNAI1
SNAI2
ETS1
B
EMT Score
High
0
Low
6
4
2
0
-2
-4
EMT Score
CDH1
CLDN7
FN1
COLVI
CDH2
ETS1
C
MUC1
TJP3
CLDN7
CLDN4
CDH1
SNAI2
SNAI1
TWIST2
TWIST1
ZEB2
ZEB1
CDH2
VIM
FN1
ETS1
EMT Score
3000
2000
1000
0
3
1.5
0
-1.5
-3
-518.56
-420.95
-379.02
-249.10
-88.68
248.16
456.50
3559.97
EMT Score
SCC47
SCC23
SCC29
HSC3
Cal27
SCC103
SCC4
SCC25
